# Cellular senescence induced by cholesterol accumulation is mediated by lysosomal ABCA1 in APOE4 and AD

**DOI:** 10.1186/s13024-025-00802-7

**Published:** 2025-02-04

**Authors:** Shaowei Wang, Boyang Li, Jie Li, Zhiheng Cai, Cristelle Hugo, Yi Sun, Lu Qian, Julia TCW, Helena C. Chui, Dante Dikeman, Isaac Asante, Stan G. Louie, David A. Bennett, Zoe Arvanitakis, Alan T. Remaley, Bilal E. Kerman, Hussein N. Yassine

**Affiliations:** 1 Keck School of Medicine, University of Southern California, Los Angeles, CA 90033 USA; 2 Department of Pharmacology, Physiology & Biophysics, Chobanian & Avedisian School of Medicine, Boston University, Boston, MA 02118 USA; 3 Bioinformatics Program, Faculty of Computing & Data Sciences, Boston University, Boston, MA 02215 USA; 4 Alfred E. Mann School of Pharmacy, University of Southern California, Los Angeles, CA 90089 USA; 5 Department of Ophthalmology, Keck School of Medicine, Los Angeles, CA 90033 USA; 6 Rush Alzheimer’s Disease Center, Rush University Medical Center, Chicago, IL 60612 USA; 7 National Heart, Lung and Blood Institute, National Institutes of Health, Bethesda, MD 20892 USA

**Keywords:** Alzheimer’s disease, Senescence, ABCA1, Caveolin-1, Cholesterol, Lysosome

## Abstract

**Background:**

Cellular senescence, a hallmark of aging, has been implicated in Alzheimer’s disease (AD) pathogenesis. Cholesterol accumulation is known to drive cellular senescence; however, its underlying mechanisms are not fully understood. ATP-binding cassette transporter A1 (ABCA1) plays an important role in cholesterol homeostasis, and its expression and trafficking are altered in APOE4 and AD models. However, the role of ABCA1 trafficking in cellular senescence associated with APOE4 and AD remains unclear.

**Methods:**

We examined the association between cellular senescence and ABCA1 expression in human postmortem brain samples using transcriptomic, histological, and biochemical analyses. Unbiased proteomic screening was performed to identify the proteins that mediate cellular ABCA1 trafficking. We created ABCA1 knock out cell lines and mouse models to validate the role of ABCA1 in cholesterol-induced mTORC1 activation and senescence. Additionally, we used APOE4-TR mice and induced pluripotent stem cell (iPSC) models to explore cholesterol-ABCA1-senescence pathways.

**Results:**

Transcriptomic profiling of the human dorsolateral prefrontal cortex from the Religious Order Study/Memory Aging Project (ROSMAP) cohort revealed the upregulation of cellular senescence transcriptome signatures in AD, which correlated with ABCA1 expression and oxysterol levels. Immunofluorescence and immunoblotting analyses confirmed increased lipofuscin-stained lipids and ABCA1 expression in AD brains and an association with mTOR phosphorylation. Discovery proteomics identified caveolin-1, a sensor of cellular cholesterol accumulation, as a key promoter of ABCA1 endolysosomal trafficking. Greater caveolin-1 expression was observed in APOE4-TR mouse models and AD human brains. Oxysterol induced mTORC1 activation and senescence were regulated by ABCA1 lysosomal trapping. Treatment of APOE4-TR mice with cyclodextrin reduced brain oxysterol levels, ABCA1 lysosome trapping, mTORC1 activation, and attenuated senescence and neuroinflammation markers. In human iPSC-derived astrocytes, the reduction of cholesterol by cyclodextrin attenuated inflammatory responses.

**Conclusions:**

Oxysterol accumulation in APOE4 and AD induced ABCA1 and caveolin-1 expression, contributing to lysosomal dysfunction and increased cellular senescence markers. This study provides novel insights into how cholesterol metabolism accelerates features of brain cellular senescence pathway and identifies therapeutic targets to mitigate these processes.

**Supplementary Information:**

The online version contains supplementary material available at 10.1186/s13024-025-00802-7.

## Background

Cellular senescence, defined as the permanent arrest of cell proliferation, is a key feature of aging and age-related diseases; however, little is known about cellular senescence in the brain of patients with Alzheimer’s disease (AD) [1–3]. Senescent cells secrete a range of inflammatory mediators, collectively known as the senescence-associated secretory phenotype (SASP) [4–6] that contribute to AD pathology. Cellular senescence is triggered by various factors such as DNA damage, reactive oxygen species, telomere attrition, lysosomal dysfunction, tau and amyloid aggregation, and lipid accumulation [2, 7, 8]. Senescent astrocytes, microglia, endothelial cells, and neurons have been observed in the brains of patients with AD and in AD mouse models [5, 9, 10] and are often associated with tau aggregation [11]. Senolytic therapies that target senescent cells have been shown to alleviate cognitive deficits in AD mouse models [11–13] and are safe in patients with AD [14, 15]. However, the precise drivers of cellular senescence in the brain and their relationship with neuropathological markers of AD remain poorly understood.

Apolipoprotein E4 allele (APOE4) is the strongest genetic risk factor for late-onset AD [16, 17]. Some features of increased senescence were observed in neurons from APOE4-target replacement (APOE4-TR) mice compared to those from APOE3-TR mice [18]. APOE4’s effects in the brain are partly mediated mainly by its cholesterol-transporting capabilities [19–21]. Cholesterol accumulation induces cellular senescence [22–24]. The ATP-binding cassette A1 (ABCA1), a transmembrane protein, plays a crucial role in transporting intracellular free cholesterol to lipidate ApoE and form HDL-like particles for cholesterol transport in the brain [25–28]. Recycling of ABCA1 to the plasma membrane is critical for cholesterol efflux into different apolipoproteins within brain cells [29]. Oxidized cholesterol species, also known as oxysterols, are potent ABCA1 inducers [30]. We previously reported that APOE4 impairs ABCA1 recycling and promotes its trafficking to lysosomes in astrocytes [31]. Lysosomal ABCA1 is necessary for inducing SASP in senescent fibroblasts, rerouting cholesterol to lysosomes to promote mTORC1 scaffolding complex activation [7].

The relationship between cell-specific senescence signatures in the brain with ABCA1 expression and AD neuropathology remains unclear. To address this gap, we analyzed cellular senescence signatures using bulk-RNA and single-nucleus RNA sequencing (snRNA-seq) of postmortem human brain tissues obtained through the Religious Order Study/Memory Aging Project (ROSMAP). We measured some of the senescence markers together with ABCA1 expression in postmortem human brain tissue samples with and without AD across different APOE genotypes. In addition, we explored the mechanisms of ABCA1 trafficking using cell lines, primary cells, human induced pluripotent stem cells (iPSC), and humanized APOE-TR mouse models.

## Methods

### Study design and sample information

To determine whether cellular senescence is associated with ABCA1 expression in APOE4 and AD, we analyzed the databases of bulk-RNA sequencing from 632 participants and single-nucleus RNA sequencing from 427 participants, all derived from the dorsolateral prefrontal cortex (DLPFC) of postmortem human brain samples in the Religious Orders Study or the Rush Memory and Aging Project (ROSMAP). Clinical and pathological characteristics of the samples are detailed in Supplementarys Table (1) and Table (2). Validation of the RNA-sequencing findings was performed using frozen mid-frontal lobe brain tissues from 138 participants and formalin-fixed paraffin-embedded (FFPE) slides from 11 participants. To identify the proteins that regulate ABCA1 degradation, proteomics was conducted, with ABCA1 purified from HeLa cells overexpressing ABCA1 and treated with recombinant ApoE3 or ApoE4 proteins. The results from the proteomics were further validated through biochemistry and histochemistry analyses. KEGG pathway analysis of 768 shared proteins was performed using the Database for Annotation, Visualization, and Integrated Discovery (DAVID) platform [32], and identified several pathways mediating ABCA1 degradation. To evaluate the impact of cholesterol reduction, 14–15 APOE4-TR mice per group were subjected to behavioral tests and treatments, with testers blinded to group allocation. All animal experiments were approved by the Institutional Animal Care and Use Committee of the University of Southern California, ensuring minimal animal stress and use. For all experiments, the number of replicates, statistical tests, and P values are reported in the figure legends.

### Analysis of bulk-RNA sequencing data

#### Database

Filtered raw counts were downloaded from the Synapse AD Knowledge Portal (https://www.synapse.org/#!Synapse:syn9702085) with Synapse ID: syn8456637.

Source data were collected from a cohort of 632 subjects from ROSMAP [33]. Both studies were approved by the Institutional Review Board of Rush University Medical Center. All participants signed informed and repository consent forms and the Anatomical Gift Act. Based on clinical diagnosis at the time of death, 83 study participants were diagnosed with no cognitive impairment (NCI), 162 with mild cognitive impairment (MCI), and 151 with AD dementia [34–36]. CERAD Braak staging represents the amount and distribution of neuritic plaques and neurofibrillary tangle pathology [37, 38]. APOE genotype was determined as previously described [39]. Detailed information regarding all samples is provided in Supplementary Table 1.

### Gene set enrichment analysis (GSEA)

The R package GSVA (method option “ssGSEA”) was used to calculate the pathway enrichment score of each subject. The annotated gene sets were retrieved from MsigDB. REACTOME_CELLULAR_SENESCENCE (M27188) was selected to represent cellular senescence. The pathway score was tested to determine the differences between the variables of interest.

### Analysis of single nucleus-RNA sequencing data

#### Database

Post-QC counts and ROSMAP metadata were downloaded from the Synapse AD Knowledge Portal (https://www.synapse.org/#!Synapse:syn52293417) with Synapse ID: syn2580853. Source data were collected from a sample of 427 subjects from ROSMAP [40]. Detailed information about the samples is provided in Supplementary Table 2. Nuclei were isolated from frozen postmortem brain tissues and subjected to droplet-based single-nucleus RNA sequencing (snRNA-seq).

### Gene set enrichment analysis (GSEA)

The R package GSVA (method option “ssGSEA”) was used to calculate the pathway enrichment score of each cell. Single sample GSEA (ssGSEA) is a non-parametric method that calculates the normalized difference in empirical cumulative distribution functions (CDFs) of gene expression ranks inside and outside the gene set, representing the enrichment score for the gene set. The annotated gene sets were retrieved from MsigDB. REACTOME_CELLULAR_SENESCENCE (M27188) and REACTOME_SENESCENCE_ASSOCIATED_SECRETORY_PHENOTYPE_SASP (M27187) were selected to represent cellular senescence and SASP. The linear mixed effect model was used to test if the pathway score was different between the variables of interest, using the formula: ~ size factor (i.e. the library size of each cell) + interested variables + (1|projid (i.e. sample id)).

### SenTraGor (STG) staining

The formalin-fixed paraffin-embedded (PPFE) human brain slides containing middle frontal lobe region (ROS) were deparaffinized following the protocol (xylene, 3 min each time for two times; xylene 1:1 with 100% ethanol, 3 min each time for two times; 100% ethanol, 3 min each time for two times; 95% ethanol, 3 min each time for two times; 70% ethanol, 3 min each time for two times; 50% ethanol, 3 min each time for two times; running cold tap water to rinse for 5 min). The slides were then subjected to antigen retrieval using a Sodium Citrate buffer (10 mM Sodium Citrate, 0.05% Tween 20, pH 6.0) for 30 min. The slides were washed once with TBS for 5 min and incubated with STG (Bio-Techne, #7555, 2 mM, dissolved in 100% ethanol, filtered with 0.45 µM filter) for 10 min at room temperature (RT). After incubation, the slides were washed twice with 50% ethanol followed by one time wash with TBST (0.025% Triton X-100 in TBS) for 5 min each wash. Then, primary antibodies (goat anti-biotin and anti-GFAP) were added to the slides and incubated overnight at 4 °C. Negative control was set as without goat anti-biotin antibody or anti-GFAP antibody. After washing three times with TBST, the slides were incubated with fluorescein-conjugated secondary antibodies at room temperature for 1 h. After washing three times with TBST, the slides were incubated with an autofluorescence quencher at room temperature for 5 min. After washing thrice with TBST, the slides were mounted with mounting medium (F4680; Sigma). Images were acquired using Leica SP8 confocal microscope (63x objective) and analyzed using the ImageJ software (NIH). The slide order number was used in image acquisition and analysis to blind the disease groups or genotypes.

### Human brain tissue homogenate preparation

Frozen mid-frontal lobes of postmortem human brains were obtained from the Rush Alzheimer’s Disease Center (RADC) at the Rush University Medical Center. The Religious Orders Study (ROS) was approved by the Institutional Review Board (IRB) of Rush University Medical Center [41]. Detailed information on these samples is presented in Supplementary Table 3.

To extract soluble and insoluble proteins, frozen middle frontal lobe brain tissues were weighed and homogenized with TBS (1:15, w/v) containing a protease inhibitor cocktail and phosphatase inhibitor cocktail. The homogenate was centrifuged at 15,000 x g for 1 h at 4 °C. The supernatant was collected as the TBS fraction, and the pellets were incubated with TBSX (1% Triton X-100) (same volume as TBS) overnight with agitation at 4 °C. The next day, the homogenate was centrifuged at 15, 000 × g for 1 h at 4 °C and the supernatant was collected as the TBSX fraction. The protein concentrations of all fractions were measured using a bicinchoninic acid kit and used for further experiments. For ABCA1 detection, TBSX fraction samples were added to 4x Laemmli sample buffer (without boiling) and subjected to 12% SDS-PAGE for western blotting.

### Lysosome enrichment with LAMP-2 immunoprecipitation

LAMP-2 antibody (1.2 µg) was added to the TBSX fraction samples (300µL) of AD E3/3 and AD E3/4 individuals. After incubation for 1 h at 4 °C, 20 µL Dynabeads Protein A/G (88802; Thermo Scientific) was added and incubated with rotation overnight at 4 °C. After two washes with 1 mL of ice-cold 0.1% TBST, 20 µL of elution buffer (50 mM Glycine, pH2.8) was added and incubated for 2 min at room temperature. The elution was quickly neutralized with 2 µL of Tris buffer (1 M, pH 8.5). Then LAMP-2 enriched samples were added to 4x Laemmli sample buffer (without boiling) and loaded onto a 7.5% SDS-PAGE gel for western blotting.

### ABCA1 complex purification

HeLa cells expressing ABCA1-GFP were cultured in DMEM (Corning, 10–013) with 10% fetal bovine serum (FBS) (Omega Scientific, FB-12) and 1% antibiotic-antimycotic (anti-anti) (Thermo Fisher, 15240062) at 37 °C in a 5% CO_2_ incubator. When the cells were ready, recombinant APOE3 or APOE4 (0.2 µM) was added for 4 h. After treatment, the cells were scratched, collected with DPBS (Corning, 21-031-CV), and washed twice with ice-cold DPBS. Then, 5 volumes of lysis buffer (0.5% NP40, 350 mM Nacl, 20 mM Hepes, 1.2% Triton X-100, complete cocktail) were added and incubated with rotation for 5 min at 4 °C. The cell lysate was centrifuged for 15 min at 13,000 rpm and 4 °C, and the supernatants were collected for immunoprecipitation.

100 µL of GFP-Trap Agarose (gta-20, Chromotek) was first washed with IP buffer (PBS 7.5–7.9; 200 mM NaCl, 1 mM EDTA, 0.5% Triton X-100, 1 mM DTT and 0.2 mM PMSF) three times and then added to 2 mL supernatant of HeLa (ABCA1-GFP) cell lysates and incubated with rotation overnight at 4 °C. The next day, agarose was washed with IP buffer three times with rotation at 4 °C. After washing, a 2-fold volume of glycine (0.1 M, pH 2.7) was used to elute the ABCA1 complex and neutralized with 1/10 volume of Tris-HCl (1 M, pH 9.5) after elution. Parts of elution were used for SDS-PAGE analysis, and other parts were added with TCA (SA433-500, Fisher Scientific) to a final concentration of 20% and incubated for 15 min to precipitate proteins. After centrifugation (13,000 rpm, 4 °C, 30 min), the pellet was collected and washed once with 1mL of acetone (A949-1, Fisher Scientific). After removing the acetone, the protein pellets were airdry for 3–4 min at room temperature to ensure that no liquid remained. Finally, the protein pellet was sent to a mass spectrometry facility (Harvard Center for Mass Spectrometry).

For ABCA1 degradation experiments, Hela cells expressing ABCA1-GFP were treated with recombinant APOE3 or APOE4 (0.2 µM) (Academy Biomedical Company) for different time points. After treatment, cells were lysed with radioimmunoprecipitation assay (RIPA) buffer (9806, Cell Signaling Technology, CST), and total ABCA1 protein levels were detected by western blotting.

### Analysis of mass spectrometry data

Normalized Spectral Abundance Factors (NSAFs) [42] were used to measure the relative abundance and calculate the fold change for each protein. To obtain the NSAF value for a specific protein, the spectral abundance factor (SAF) value was first calculated by dividing the spectral count of the protein by its amino acid length. The NSAF was then calculated by normalizing the SAF value to the total SAF values of all the identified proteins within each sample. This approach allows for the accurate assessment of protein abundance across different samples.

### Immunocytochemistry

Immortalized astrocytes were grown in 8-well cell culture chamber slides (Bioland Scientific, 07-2108). After fixing with 4% paraformaldehyde for 15 min, the cells were permeabilized with 1% Triton X-100 for 30 min and then blocked with 10% goat serum and 0.1% Triton X-100 for 1 h at room temperature. After blocking, the cells were incubated with anti-ABCA1 antibody (Abcam, ab18180) (1:100) and anti-Caveolin-1 antibody (CST, 3267 S) (1:200) or anti-AP2B1 antibody (GeneTex, GTX79316) (1:200) overnight at 4 °C. After washing, the cells were incubated with Alexa Fluor Plus 488 or 594 labelled Goat anti-Mouse IgG (Thermo Fisher, A32723/A-11005, 1:200) and Alexa Fluor 594 or 488 labelled Donkey anti-Rabbit IgG (Thermo Fisher, A21207/A-21206) (1:200) for 1 h at room temperature in the dark. After washing, the nuclei were stained with Hoechst 33,258 (Thermo Fisher, H3569) (1:12,000) for 5 min. After washing, the slides were mounted with mounting medium (F4680; Sigma). Images were captured using a Zeiss Axiovert 200 M Inverted Fluorescence Microscope (20x objective). Images were analyzed using ImageJ software (NIH).

### Cell culture

Primary astrocytes were obtained from APOE3-TR and APOE4-TR mouse pups and cultured as previously described [43]. Briefly, cerebral cortices from 1 to 3-day-old neonatal mice were dissected in ice-cold Hanks’ balanced salt solution (HBSS) (Corning, 21-021-CV) and digested with 0.25% trypsin for 20 min at 37 °C. Trypsinization was stopped by adding a 2-fold volume of DMEM (Corning, 10–013) with 10% fetal bovine serum (FBS) (Omega Scientific, FB-12) and 1% antibiotic-antimycotic (anti-anti) (Thermo Fisher, 15240062). The cells were dispersed to a single-cell level by repeated pipetting and filtered through 100 μm cell strainers (VWR, 10199-658). After filtering, the cells were centrifuged for 5 min at 1000 rpm and resuspended in culture medium (DMEM, Corning, 10–013) supplemented with 10% FBS and antibiotics. The cells were then seeded in a 75 cm^2^ flask and cultured at 37 °C in 5% CO2. The medium was changed the next day and replaced every three days. The mixed glial cultures reached confluence after 7–10 days. The cells were then shaken at 250 rpm for 16 h at 37 °C to remove the microglia and oligodendrocyte progenitor cells. The remaining cells were harvested by trypsin digestion. At this stage, the culture contained 95% astrocytes, which were used for further experiments.

Immortalized mouse astrocytes derived from human APOE3 and APOE4 knock-in mice were gifts from Dr. David Holtzman and grown in DMEM/F12 (Corning, MT10090CV) containing 10% FBS, 1mM sodium pyruvate (Thermo Fisher, 11360070), 1 mM geneticin (Thermo Fisher, 10131-035) and 1% anti-anti.

### Cell plasma membrane protein preparation

To study cell plasma membrane proteins, biotinylation of cell surface proteins was performed to isolated cell plasma membrane protein. Briefly, cells were washed twice with cold PBS followed by incubation with 0.5 mg/ml sulfo-NHS-SS-biotin (Thermo Fisher Scientific, PG82077) in PBS for 30 min at 4 °C with shaking. The reaction was quenched by rinsing cells with 50 mM glycine in PBS. The cells were then lysed with RIPA buffer (CST, 9806) containing a protease inhibitor cocktail, followed by centrifugation at 12,000 × g for 10 min at 4 °C. The supernatant was collected, and protein concentrations were measured using a BCA kit (Thermo Fisher, 23225). The radioimmunoprecipitation assay (RIPA) fraction represented the total protein fraction. Then, 100 µg of the RIPA fraction from each sample was incubated with 40µl of NeutrAvidin agarose (Thermo Fisher Scientific, 29200) for 2 h at 4 °C. The agarose was then washed three times with PBS containing a protease inhibitor cocktail, followed by boiling with 100 µL 2x sample buffer for 5 min at 95 °C. After centrifugation (2,500 x g, 2 min, room temperature), the supernatants were collected as plasma membrane proteins. Membrane and total protein analyses were performed using western blotting.

### Immunoprecipitation

HeLa (negative) or HeLa (ABCA1-GFP) cells were lysed in RIPA buffer containing protease and phosphatase inhibitors. 10 µL of GFP-Trap Agarose was added to 200 µL cell lysates (1 µg/µL) and incubated with rotation overnight at 4 °C. The next day, agarose was washed with IP buffer three times with rotation at 4 °C. After washing, a 2-fold volume of diluted (2x) sample buffer (Bio-Rad, 1610747) was added and boiled for 5 min at 95 °C. After centrifugation (1,500 rpm, 5 min, room temperature), supernatants were collected for SDS-PAGE and western blotting.

Astrocytes were lysed in radioimmunoprecipitation assay (RIPA) buffer containing protease and phosphatase inhibitors. 1.5 µg antibody (anti-ABCA1) was added to 400 µL cell lysates (1 µg/µL) and incubated with rotation for 1 h at 4 °C. Then, 25 µL Dynabeads Protein G (Thermo Scientific, 10004D) was added and incubated with rotation overnight at 4 °C. After three washes with 1 mL of ice-cold 0.1% TBST, 25 µL of diluted (1.5 ×) sample buffer was added to the beads and boiled for 5 min at 95 °C. The supernatants were collected and subjected to SDS-PAGE and western blotting. 

### Western blotting

The cell lysates, brain homogenates, or immunoprecipitation protein complexes were separated on 4–15% mini-precast protein gels (Bio-Rad, 4561086) under reducing conditions and then transferred onto nitrocellulose membranes (Bio-Rad, 1704270). After transfer, the membranes were blocked with 5% fat-free milk (Bio-Rad, 1706404) in TBST for 1 h at room temperature, followed by overnight incubation with the primary antibody in 5% BSA at 4 °C. The membranes were then incubated with an HRP-conjugated secondary antibody for 1 h at room temperature. A chemiluminescent HRP substrate (Millipore, WBKLS0500) was used for detection. The Fujifilm LAS-4000 imager system or X-ray film was used to capture images, and densitometric quantification was performed using Gel Quant NET software. The antibodies used are listed in Supplementary Table 5.

### siRNA and plasmid transfections

Non-targeted (NT), Caveolin-1 and AP2B1 siRNAs were obtained from Dharmacon. Cells were seeded in 60 mm dishes or 24-well plates and cultured overnight. The siRNAs (10–20 nM) were transfected using the jetPRIME reagent (Polyplus Transfection, catalog #114). Protein expression was detected using western blotting 48 h after transfection.

The mouse Caveolin-1 (Myc-DDK-tagged) expression plasmid (MR201562) was purchased from Origene and purified using an endo-free plasmid DNA midi kit (Bioland Scientific, PD03-21). The pCMV-GFP expression plasmid was used as the control. The cells were seeded in 24-well plates and cultured overnight. The plasmid (0.125 µg) was transfected using the jetPRIME reagent. Protein expression was detected using western blotting 48 h after transfection.

### Cholesterol efflux

Immortalized astrocytes were seeded in 24-well plate (0.12 × 10^5^ cells/500 µL) and cultured overnight. The cells were transfected with 20nM siRNA for 24 h, followed by labeling with 1µCi/mL (3 H) cholesterol (Moravek, MT9112) using serum-free DMEM/F12 containing 2 mg/ml fatty acid-free BSA (Sigma-Aldrich, catalog #A9647), and 2 µg/ml acyl-coenzymeA: cholesterol acyltransferase inhibitor SANDOZ (Sigma-Aldrich, catalog #S9318) for 24 h. After washing once with serum-free culture medium, the cells were treated with recombinant CS-6253 peptide (1 µM in DMEM/F12 containing 2 mg/mL fatty acid-free BSA, 2 µg/mL SANDOZ, and 200 µL/well) for 4 h. After treatment, the cell culture medium was collected and transferred to scintillation vials filled with 3 mL of the scintillation mixture. The cells were solubilized in 0.5 N NaOH (200 µL), neutralized with PBS, and then transferred to scintillation vials filled with 3 mL of scintillation mixture. After vigorous mixing, vials were counted using a Beckman LS6500 liquid scintillation counter (Beckman Coulter). The efflux of cholesterol was assessed by the ratio of cholesterol in the medium to the total cholesterol (medium and cell lysate).

### Cholesterol loading and depletion

Immortalized astrocytes or baby hamster kidney (BHK) cells were seeded in 24-well plates (0.5 × 10^5^ cells/500 µL) and cultured overnight. Cells were washed twice with serum-free medium, and human LDL (10 µg/mL diluted in serum-free medium containing 2 mg/mL fatty acid-free BSA) (Sigma, LP2-2MG) was added for 24 h. The cholesterol levels in the cells were determined using a cholesterol assay kit (filipin III staining, Abcam, ab133116). Cellular caveolin 1 protein levels were determined by western blotting. Human iPSC astrocytes were seeded in 48-well plates (0.5 × 105 cell/300µL) and cultured for 48 h. Cells were first loaded with LDL as described above and treated with 1 mM methyl-β-cyclodextrin (MβCD) for 2 h. Cellular cholesterol levels were determined using filipin staining. For parallel experiments, after MβCD treatment, cells were stimulated with TNFα (100 ng/mL) and IFNγ (100 ng/mL) for 16 h. RNA was extracted from each condition using an RNA extraction kit (Thermo Fisher, K0731).

### ABCA1 knock down cell line and cholesterol loading

An ABCA1 knockdown astrocyte cell line was created by transfection with ABCA1 CRISPR Gene Knockout Kit (ABCA1-sgRNAs and Cas9 recombinant protein, Synthego). Three days after transfection, the single cell was seeded and cultured for two weeks. ABCA1 knockout or knockdown clones were validated by western blotting using ABCA1 antibody. ABCA1 WT or ABCA1 knockdown astrocytes were seeded in 24-well plates (0.5 × 10^5^ cell/500 µL) and cultured overnight. Cells were treated with methyl-β-cyclodextrin (MβCD, 5 mg/mL) for 2 h and then loaded with LDL (100 µg/mL) for 2 h. Then, the cells were lysed for further analysis.

### ABCA1 induction and cholesterol loading in BHK cells

BHK cells were seeded in 24-well plates (0.5 × 10^5^ cell/500 µL) and cultured overnight. ABCA1 expression was induced with Mifepristone (0.1 nM) treatment for 16 h. Then, cells with or without ABCA1 were loaded with LDL (100 µg/mL) for 24 h. The cells were lysed for further analysis.

### Animals and treatment

All animal experiments were approved by the Institutional Animal Care and Use Committee of the University of Southern California. Animals were housed under a standard 12-hour light/dark cycle with water and chow diet in a pathogen-free animal facility at the University of Southern California. Every effort was made to reduce animal stress and minimize animal use. ABCA1^fl/fl^ mice were obtained from Dr. John Parks of Wake Forest University School of Medicine. Nestin-cre mice (Strain #:003771) were purchased from Jax. ABCA1 conditional knockout mice were generated by crossing ABCA1^fl/fl^ mice with nestin-Cre mice. ABCA1 genotype was confirmed by PCR genotyping. Ten-month-old wild type mice and ABCA1 conditional knockout mice were euthanized with CO_2_ and perfused with cold PBS. Brains were collected for further experiments.

The APOE3-TR and APOE4-TR mice were purchased from JAX. APOE3 and APOE4-TR mice of different ages were euthanized using CO_2_ and perfused with cold PBS. Brains were collected for further experiments.

APOE4-TR male and female mice (8 months old) were randomized to receive subcutaneous injections of 2 g/kg body weight 2-hydroxypropyl-β-cyclodextrin (HPCD) (Sigma, 332607-25G) in phosphate-buffered saline (PBS) or PBS twice a week for eight weeks. The mice were then subjected to behavioral tests, and the testers were blinded to the treatment allocation. The injection was continued during behavioral tests (two weeks). Finally, after collection of the cerebrospinal fluid (CSF) and plasma, the mice were anesthetized and subjected to cardiac perfusion with ice-cold PBS. The brains were dissected, and one hemisphere was post-fixed in 4% paraformaldehyde (PFA) for cryostat sectioning, while the other was used for biochemistry assays and RNA purification.

### Novel object recognition (NOR)

This was performed as previously reported with some modifications [44]. On day 1, mice were acclimated to the test room for 1 h followed by free exploration for 5 min in the test arena (without objects). On day 2, mice were placed into the same arena containing two identical objects and allowed to freely investigate both for 5 min (training trial). After an interval of two hours, the mice were placed back in the chamber with one novel object and one familiar object and allowed to explore for 5 min (testing trial). After each trial, the testing area and objects were thoroughly cleaned with 70% ethanol solution. The training and testing trials were recorded with a high-resolution camera, and the number of touches to both objects of each mouse in the testing trial was analyzed using the EthoVision XT software (Noldus). Mice touching the object with the nose were identified as one instance of nose touch. The movement distance, total touch times for both objects, and ratio of nose touch times of the novel object to the old object were calculated. One mouse in the HPCD group was excluded because of a fighting wound.

### Cell lysate and mouse brain homogenate preparation

Immortalized or primary astrocytes were lysed with 1x RIPA buffer (CST, 9806) containing protease inhibitor cocktail (Sigma, P8340) and phosphatase inhibitor cocktail (Sigma, P0044), followed by centrifugation at 15,000 × g for 10 min at 4 °C. The supernatant was then collected for further analysis. The mouse cerebral cortex was weighed, and RIPA buffer containing a protease inhibitor cocktail and phosphatase inhibitor cocktail was added at a ratio of 1:30 (w/v). The tissue was then homogenized using a 2 mL glass Dounce tissue grinder, followed by centrifugation at 15, 000 × g for 1 h at 4 °C. The supernatant was collected, and the concentration was measured using a BCA kit.

To extract soluble and insoluble proteins, mouse and human brain samples were weighed and homogenized with TBS (1:15, w/v) containing protease and phosphatase inhibitor cocktails. The homogenate was centrifuged at 15,000 x g for 1 h at 4 °C. The supernatant was collected as the TBS fraction, and the pellets were incubated with TBSX (1% Triton X-100) (same volume as TBS) overnight with agitation. After centrifugation (15,000 × g, 1 h at 4 °C), the supernatant was collected as the TBSX fraction. The pellets were dissolved in 150–200 µL guanidinium chloride (GnHCl) (5 M, pH 7.5) and incubated overnight at room temperature with agitation. After centrifugation (15,000 × g, 1 h at 4 °C), the supernatant was collected and dialyzed with a dialysis device (MINI Dialysis Device, 3.5 K MWCO, 0.1 mL, Thermo Scientific) in TBS overnight at 4 °C. The solution was collected as the GnHCl fraction. The protein concentrations of all fractions were measured using a bicinchoninic acid kit and used for further experiments.

### Immunohistochemistry and immunofluorescence staining of mouse brain slides

Fixed mouse brains were sectioned using a Leica cryostat at a thickness of 10 μm. Slides were equilibrated for 30 min at room temperature and rinsed twice with PBS. After blocking with blocking buffer (10% goat serum + 1% BSA + 0.2% Triton X-100 in PBS) at room temperature for 1 h, the sections were incubated with diluted primary antibodies (GFAP, Iba-1) and incubated overnight at 4 °C. After washing, the slides were incubated in a 0.3% H_2_O_2_ solution in PBS at room temperature for 10 min to block endogenous peroxidase activity. Then, the SignalStain^®^ Boost IHC Detection Reagent (#8114, Cell Signaling Technology) was added to the slides and incubated at room temperature for 1 h. After washing, DAB substrate solution was added to the slides and developed for 2–5 min until the desired color intensity was reached. After washing, the slides were mounted with mounting medium (F4680; Sigma). Images were taken using an Olympus Microscope (10x objective). Images were analyzed using the ImageJ software (NIH).

For ABCA1 and LAMP1 double staining, after overnight incubation with the primary antibody (ABCA1, Abcam, ab18180; LAMP1, CST, #99437), the fluorescein labelled secondary antibody was added to the slides and incubated at room temperature for 1 h (protected from light). After washing, the slides were mounted with mounting medium with DAPI (Sigma, F6057). Images were taken using Leica SP8 confocal microscope (63x objective) or a slide scanner (Z1, 20x objective, Zeiss Axio Scan, Oberkochen, Germany) and analyzed using the ImageJ software (NIH). For p21 and pH2A.X staining, after overnight incubation with the primary antibody (p21, CST, #37543; pH2A.X, CST, #9718), the ABC kit (Vector Laboratory, PK-4001) and Cyanine 3 tyramide (Bio-techne, #6457) were performed to obtain the fluorescence signals. After washing, the slides were mounted with mounting medium with DAPI. Images were taken using Leica SP8 confocal microscope (63x objective) and analyzed using the ImageJ software (NIH). The slide order number was used in image acquisition and analysis to blind the groups or genotypes.

### Lysosome isolation

Thirty to fifty mg of brain tissues were used to isolate lysosomes with the lysosome isolation kit (Minute Lysosome Isolation Kit, Invent Biotechnologies, LY-034) following the manuals. Briefly, the tissues were homogenate with a plastic rod for 1 min, followed by the centrifugation at 16,000 x g for 30 s. The filter was discarded, and the pellet was resuspended by vigorously vertexing for 10 s, followed by centrifugation at 2,000 x g for 3 min. The supernatant was collected to a fresh 1.5 mL microfuge tube and centrifuged at 4 °C for 15 min at 9,000 x g. After centrifugation, the supernatant was carefully transfer to a fresh 1.5 mL tube and spined at 16,000 x g at 4 °C for 30 min. After centrifugation, the supernatant was removed completely. Then, the pellet was resuspended in 200 µL of cold buffer A by pipetting up and down 60–100 times and vortex vigorously for 20 s, followed by centrifugation at 2,000 x g for 4 min. The supernatant was transferred to a fresh 1.5 mL tube and added 100 µL of buffer B, and vortexed briefly vortex to mix well (the supernatant to buffer B ratio is 2:1). The mixture was incubated on ice for 30 min and centrifuged at 11,000 x g for 10 min. After removing all the supernatant, the pellet (lysosomes) was resuspended in 50 µL of RIPA buffer for further analysis.

### qPCR

The cells and brain specimens were harvested, and RNA was extracted using an RNA extraction kit (Thermo Fisher, K0731). cDNA synthesis was performed using the High-Capacity cDNA Reverse Transcription Kit (Thermo Fisher, 4368814). qPCR was performed using the 2x SYBR Green I-based qPCR master mix (Bioland Scientific, QP01-00) on Siemens VERSANT kPCR Molecular System. The primers were listed in Supplementary Table 6. All primers were synthesized by Integrated DNA Technologies. The Ct value of the targets was normalized to 18sRNA.

### Isolation of cell types from mouse brain

Cell types isolation from mouse brain was performed following the published protocol [45]. Briefly, mice were euthanized with CO_2_ and perfused with PBS. The brains were harvest and cerebra were collected. Then, the cerebra were dissociated in the enzyme digestion mix (collagenase A (Sigma, 10103586001) + DNase I (Sigma, 10104159001) by passing three separate flame-polished Pasteur pipets to a single cell suspension. After centrifugation, the debris/myelin was removed by percoll (Sigma, GE17-0891-02) gradient centrifugation. The myelin fraction and total cells mixture were collected separately. Microglia from total cells mixture were purified by incubating with CD11b microbeads (Miltenyi Biotec,130-093-636) and passing LS magnetic column (Miltenyi Biotec, 130-042-401). CD11b negative population were also collected. Myelin was separated using gradient centrifugation. After collection, the different cell types were lysed with RIPA for western blotting detection.

### Ion-mobility analysis

HDL particles in mouse CSF and plasma were determined by ion mobility analysis as previously reported [46]. Samples treated with dextran sulfate were introduced into a charge-reducing electrospray (TSI Inc., model 3482) every 13 min by automated loop injections using an integrated autosampler (Teledyne CETAC Technologies, model MVX-7100). The electrospray settings were as follows: voltage 2.0 kV, CO2 flow 0.15 slmp, and airflow 1.5 slmp. The differential mobility analyzer (TSI Inc., model 3085), coupled to a condensation particle counter (TSI Inc, model 3788), scanned particles 4.45 to 63.8 nm for 180 s. The generated data were analyzed using Fityk (version 1.3.1), as previously described, and graphed using OriginPro software (version 2021). Voigt probability distribution curves were generated from particle count (#/mL) vs. diameter range for lipoprotein subclasses and normalized by dividing subclasses by the sum of peak areas from all lipoproteins present within the spectrum.

### Oxysterol measurement by LC-MS/MS

The Oxysterol Derivatization MaxSpec Kit and oxysterol standards were obtained from Cayman Chemical Company. Brain tissues were weighed and homogenized with steel beads in ethanol. After centrifugation at 10,000 x g for 10 min at 4 °C, 800 µL of supernatant was transferred to fresh tubes and diluted with water to a final concentration 20% ethanol. The diluted samples containing oxysterols were then extracted using Oasis HLB SPE cartridges (60 mg) that were conditioned with methanol (1 mL) and water (1 mL). Oxysterols were eluted using 70% ethanol (1 mL) and eluted again using the same procedure where the combined samples were evaporated to dryness using a steady stream of N_2_ gas. The dried oxysterol fractions were derivatized with Girard’s reagent P according to manufacturer’s instruction using an Oxysterol Derivatization MaxSpec Kit (Cayman Chemical, #601540). 5 µL was injected to an Agilent 1100 HPLC linked to API4000 mass spectrometer (Sciex). A Poroshell 120 EC-C18 column (2.7 μm, 4.6 × 100 mm, Agilent) was used for chromatographic separation. The mobile phase consisted of water + 0.1% formic acid (A) and methanol + 0.1% formic acid (B) with the following gradient: 0 min 50% B; 7 min 98% B; 16 min 98% B; 16.1 min 50%B; 20 min 50%B. Flow rate was set at 500 µL/min and column temperature was maintained at 40 °C. The MS was operated in positive ESI mode and the target analytes were quantified using their optimized MRM transition. Peak integrations were manually reviewed using Skyline software (v. 23.1, MacCoss Lab) and peak areas were normalized by tissue weight. The internal standard, 7β-hydroxy Cholesterol-d7, was used a corrective factor for sample processing and recovery. The corresponding GP-d5 analytes were used as a corrective factor to account for variance in injection volume and ionization efficiency for each sample and to confirm analyte retention times.

### Generation of human iPSC-astrocytes

APOE isogenic human iPSC–derived neural progenitor cells (NPC) were generated in the TCW laboratory [47]. Dissociated forebrain NPCs were differentiated into astrocytes in astrocyte medium (#1801, ScienCell), as previously described [48]. Briefly, forebrain NPCs were maintained at a high density on poly L-rrnithine hydrobromide (#P3655-50MG, Sigma) and laminin (#23017015, Thermo Fisher)-coated plates and cultured in NPC medium [DMEM/F12 (Corning, MT10090CV), 1 x N2 supplement (Thermo Fisher, 17502048), 1xB27 supplement (Thermo Fisher, 12587010), 1 mg/mL laminin, and 20 ng/mL FGF2 (Thermo Fisher 13256029)]. The cells were split at approximately 1:3 to 1:4 every week with Accutase (Sigma, SCR005). NPCs were differentiated into astrocytes by seeding dissociated single cells at a density of 15, 000 cells/cm^2^ on Matrigel (Corning, 356255) -coated plates and cultured in complete astrocyte medium (#1801, ScienCell). After 30 days of differentiation, the astrocytes were ready for further experiments.

### Statistical analysis

GraphPad Prism software (version 10) or R Program (regression analysis, version 4.3.2) was used for all statistical analyses. Unpaired t-test, one-way or two-way analysis of variance (ANOVA) were used to determine statistical significance, followed by Tukey’s test for multiple comparisons. All quantitative data are presented as mean ± SD. Statistical significance was defined as *p < 0.05, **p < 0.01, ***p < 0.001, ****p < 0.0001.

## Results

### ABCA1 expression is associated with markers of cellular senescence in postmortem brain cells

To determine whether cellular senescence is associated with ABCA1 expression in APOE4 and AD, we first analyzed bulk-RNA sequencing data from the dorsolateral prefrontal cortex (DLPFC) of postmortem human brain samples from 632 participants in ROSMAP. The sample included 261 APOE3 controls (APOE3/3) and 96 APOE4 carriers (APOE3/4 and APOE4/4). Based on the clinical diagnosis using clinical data collected over time and proximate to the time of death, 83 participants were classified as having no cognitive impairment (NCI), 162 as having mild cognitive impairment (MCI), and 151 as having AD dementia. A total of 164 participants were classified into Braak Stages 0, I, II, and III, 116 into Braak stage IV, and another 116 into Braak stages V and VI. The CERAD score, a semiquantitative measure of neuritic plaques, was used to classify patients as neuritic plaque-positive (*n = 241*; scores 1 and 2) and plaque-negative (*n = 155*; scores 3 and 4), as previously published [38].

**Fig. 1:**
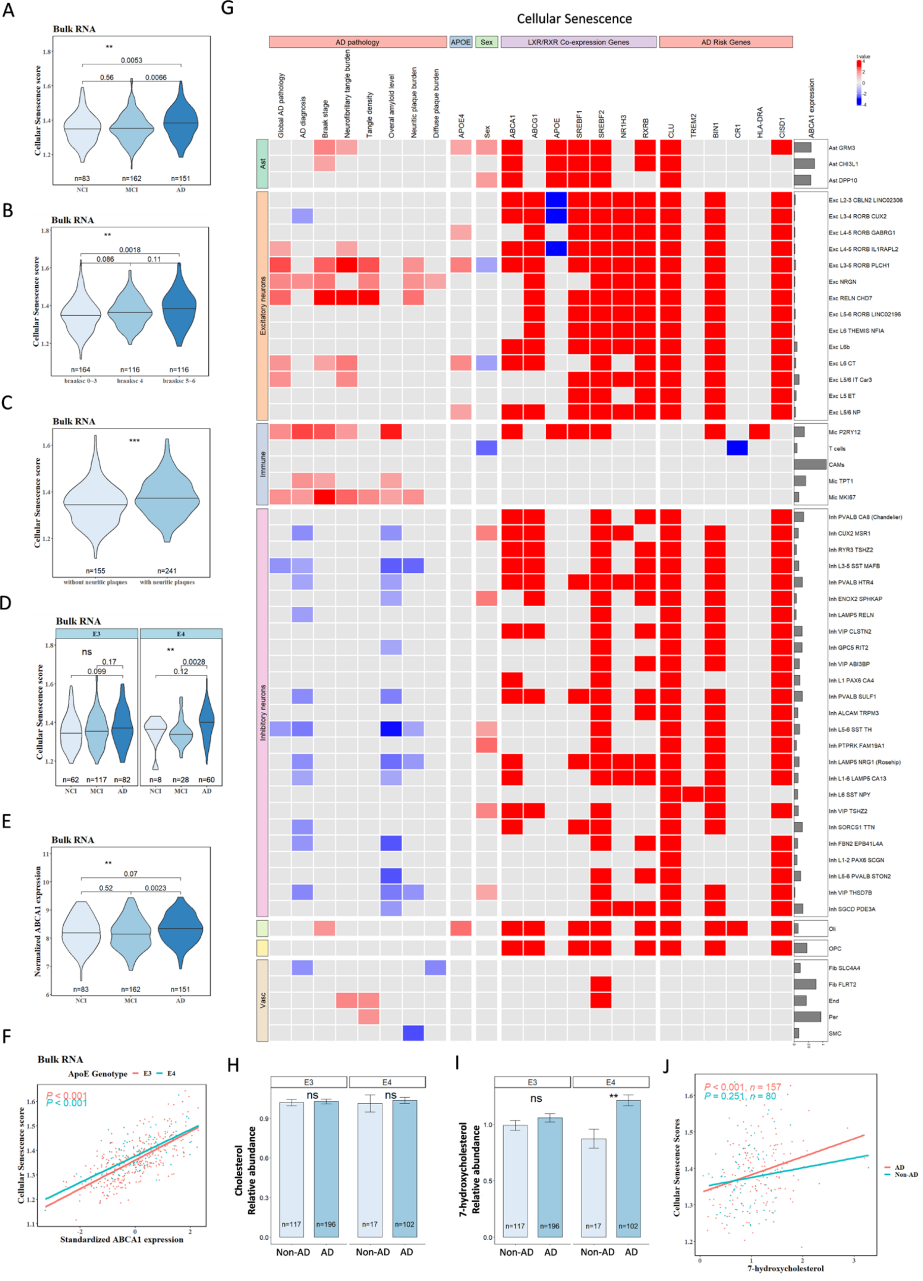
Bulk-RNA and single-nucleus RNA (sn-RNA) sequencing analyses reveales an association between ABCA1 and cellular senescence in APOE4 and AD. (**A**) Bulk-RNA sequencing analysis showing cellular senescence ssGSEA scores across no cognitive impairment (NCI), mild cognitive impairment (MCI) and AD. (**B**) Bulk-RNA sequencing analysis of cellular senescence ssGSEA scores by Braak stage groups (0–3; 4; 5–6). (**C**) Bulk-RNA sequencing analysis of cellular senescence ssGSEA scores in the groups with and without neuritic plaques. (**D**) Bulk-RNA sequencing analysis of cellular senescence ssGSEA scores across different APOE genotypes and clinical groups. (**E**) Bulk-RNA sequencing analysis of ABCA1 expression levels across clinical groups. (**F**) Association between ABCA1 expression and cellular senescence ssGSEA scores in the bulk-RNA sequencing, analyzed using a linear model. (**G**) Single-nucleus RNA sequencing analysis of the association of cellular senescence ssGSEA scores in different brain cells with factors, including AD pathology, sex, APOE genotype, LSX/RXR co-expression genes and other AD risk genes. The heatmap shows t value (coefficient 𝛃 divided by standard error of 𝛃), with red indicating significant positive associations, blue indicates significant negative associations, gray indicating non-significant associations, and cutoff *p* = 0.1). The gray bar plot represents ABCA1 expression levels in different cell types. (**H**) total cholesterol, (**I**) 7-hydroxycholesterol levels of the postmortem human cortex tissues from participants with or without AD and carrying different APOE genotypes. (**J**) Correlation between 7-hydroxycholesterol levels and cellular senescence ssGSEA scores. Data were analyzed using an unpaired t-test (**C**) or one-way ANOVA (**A**,** B**,** D**,** E**) or a linear model (**F**). ** *p* < 0.01, *** *p* < 0.01

Cellular senescence pathway enrichment scores for each sample were calculated using single-sample Gene Set Enrichment Analysis (ssGSEA) (the cellular senescence genes are listed in Supplementary Table 7). These scores, representing the relative expression levels of senescence-related genes, were higher in the AD group than in the NCI and MCI groups (*p = 0.0053* for AD vs. NCI; *p* = 0.0066 for AD vs. MCI; Fig. 1A). The cellular senescence score also increased with advanced Braak stage (Fig. 1B), and CERAD scores (Fig. 1C). APOE4 carriers with AD exhibited higher cellular senescence scores than APOE4 carriers without AD dementia, whereas no significant difference was observed among APOE3 carriers (Fig. 1D). ABCA1 mRNA levels were higher in the AD dementia group than in the NCI and MCI groups (Fig. 1E). A strong positive correlation was observed between cellular senescence ssGSEA scores and ABCA1 expression, independent of APOE genotype (*p < 0.001*, Fig. 1F).

To explore cellular level information, we analyzed single-nucleus (sn) RNA sequencing data from 2.3 million nuclei of 427 individuals from ROSMAP [40] (238 with AD dementia and 189 without AD dementia) in relation to AD neuropathology scores and ABCA1 expression. Participants were classified as APOE4 non-carriers (APOE2/2, APOE2/3, and APOE3/3) or APOE4 carriers (APOE2/4, APOE3/4, and APOE4/4). We tested whether cellular senescence is associated with AD characteristics (Global AD pathology: a quantitative summary of AD pathology derived from counts of three AD pathologies: neuritic plaques, diffuse plaques, and neurofibrillary tangles; Braak stage: a semiquantitative measure of distribution and severity of neurofibrillary tangle pathology; tangle density: immunohistochemistry for phosphorylated tau; overall amyloid level: immunohistochemistry for amyloid beta) and other AD risk factors, such as APOE4, sex, and ABCA1 mRNA expression. The association between cellular senescence scores and variables of interest was examined using a linear mixed effect model. The t-values are presented in a heat map (Fig. 1G) represent the coefficient 𝛃 divided by standard error of 𝛃.

Increased cellular senescence ssGSEA scores were associated with AD pathological factors such as neurofibrillary tangle burden and neuritic plaque burden across various brain cell types, including astrocytes, microglia, excitatory neurons, oligodendrocytes, and vascular cells (Fig. 1G). Sex differences were observed in astrocytes and inhibitory neurons (Fig. 1G). APOE4 was associated with higher cellular senescence scores in GRM3 (glutamate metabotropic receptor 3 positive) astrocytes, certain excitatory neuron types and oligodendrocytes (Fig. 1G). Importantly, ABCA1 expression strongly correlated with cellular senescence scores in most brain cell types, including astrocytes, excitatory neurons, microglia, inhibitory neurons, oligodendrocytes, and oligodendrocyte progenitor cells (Fig. 1G). In a few sub-cell types of microglia (TPT1, MKI67) and excitatory neurons (NRGN, RELN CHD7, and L5/6 IT Car3), markers of cellular senescence were not correlated with ABCA1 expression, but with Tau or Aβ pathologies, suggesting ABCA1 independent mechanisms for senescence exist (Fig. 1G). Additionally, ABCA1 expression strongly correlated with LXR/RXR pathway activation across various brain cell types (Fig. 1G), underscoring its significant role in cellular senescence. Other LXR/RXR co-expressed genes such as ABCG1, APOE, SERBF1, SERBF2, NR1H3, and RXRB were also strongly correlated with cellular senescence scores in most brain cell types (Fig. 1G). Among the AD risk genes, CLU, BIN1, and CISD1 were associated with cellular senescence scores, whereas TREM2, CR1, and HLA-DRA were not (Fig. 1G). ABCA1 expression also correlated with SASP scores in the same human brain samples (Supplementary Fig. 1A). Sn-RNA sequencing analysis also indicated that mTOR activation scores were positively associated with ABCA1 expression in astrocytes, excitatory and inhibitory neurons, microglia, oligodendrocytes, and oligodendrocyte progenitor cells (Supplementary Fig. 1B). AD characteristics, such as Braak stage, NFT burden, and neuritic plaque burden, were also positively correlated with mTOR activation in excitatory neurons (Supplementary Fig. 1B), where APOE4 was associated with increased mTOR expression in excitatory neurons, oligodendrocytes, and pericytes (Supplementary Fig. 1B). Taken together, these results indicate that in participants with AD dementia, particularly those with APOE4, cellular senescence scores were strongly correlated with ABCA1 expression and mTOR activation across different cell types.

Among cholesterol species, oxysterols are known to induce ABCA1 via LXR activation [30]. To explore the potential role of cholesterol or oxysterols in the association between ABCA1 expression and cellular senescence in AD, lipidomic data from the ROS study were analyzed. Total cholesterol levels did not differ significantly between the AD and non-AD groups (Fig. 1H). However, the oxysterol 7-hydroxycholesterol was significantly elevated in the AD group with the APOE3/4 genotype compared to that in the non-AD group (Fig. 1I). A positive association was found between 7-hydroxycholesterol levels and cellular senescence scores in patients with AD (*p < 0.001*; Fig. 1J). These findings suggest that ABCA1 expression and cellular senescence are closely linked in AD, particularly in APOE4 carriers, and point to the potential involvement of oxysterols and mTOR activation in AD pathology.

### Lysosomal ABCA1 proteins and senescence markers are elevated in APOE4 AD human brain tissues

**Fig. 2:**
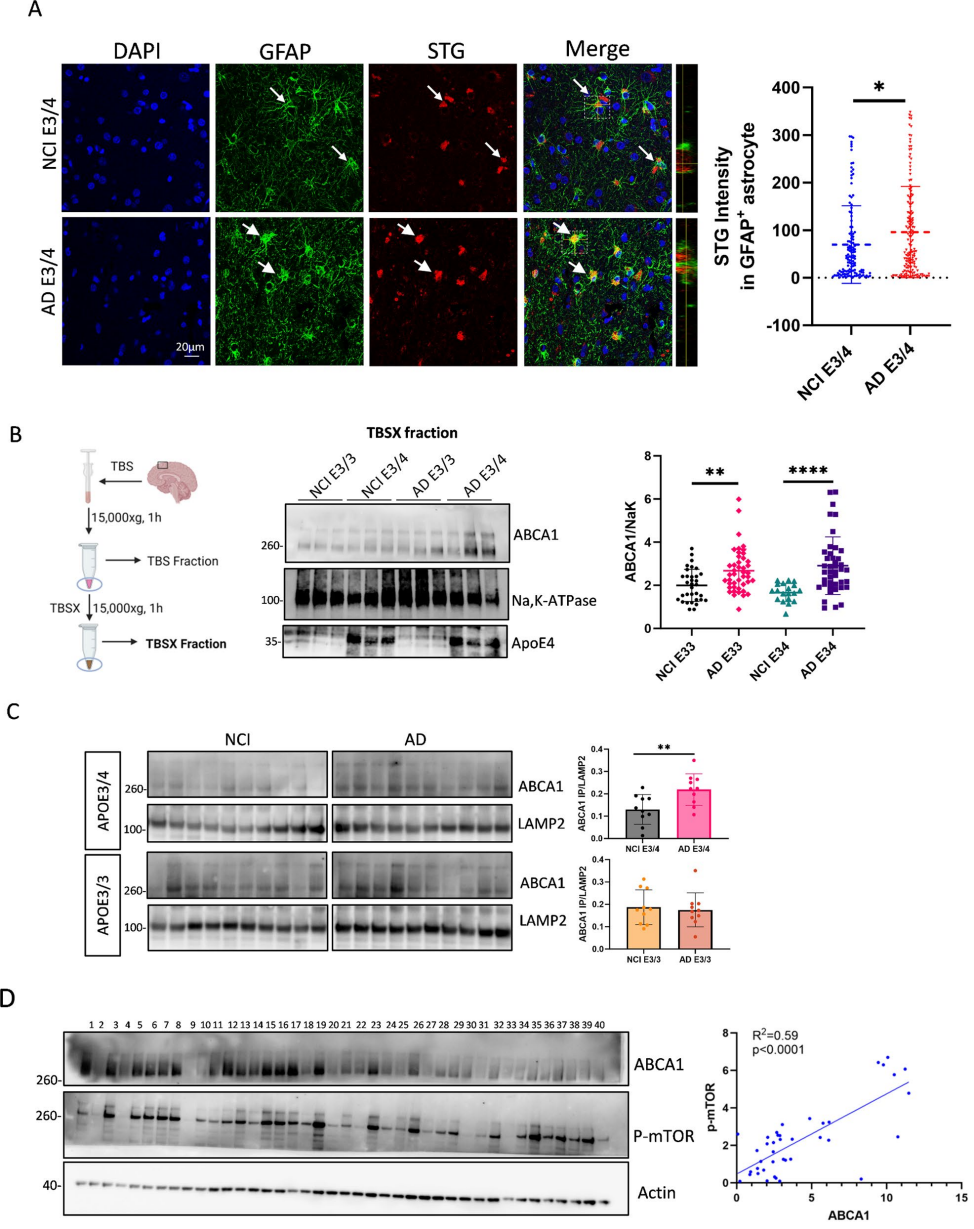
Lysosomal ABCA1 and STG are increased in AD compared to NCI in APOE3/4 carriers. All postmortem human brain tissues and slides were obtained from mid-frontal lobe tissue sections from the ROS. (**A**) Representative images showing DAPI, GFAP and STG, staining in human brain slides from AD and non-AD patients carrying APOE3/4 genotype. Arrows indicate STG signals quantified from GFAP-positive astrocytes. (*n* ≥ 126 astrocytes from 5 individuals in NCI (blue) and 6 individuals in AD (red)). (**B**) ABCA1 protein levels in total membrane extracts from human brain tissue, obtained using TBSX buffer, were detected by western blotting (WB). Quantification of total membrane ABCA1 protein levels normalized to Na,K-ATPase (a plasma membrane marker) (NCI APOE3/3, *n* = 33; NCI APOE3/4, *n* = 19; AD APOE3/3, *n* = 44; AD APOE3/4, *n* = 42). (**C**) WB blot analysis of ABCA1 and LAMP2, with LAMP2 immunoprecipitated (IP) from homogenates of human brain tissues (ABCA1 IP / LAMP2; *n* = 10 per group). (**D**) WB analysis of phosphorylated mTOR and ABCA1 protein levels in human brain tissue homogenates, with association between phosphorylated mTOR and ABCA1 protein levels analyzed using simple linear regression (*n* = 40). Data are represented as mean ± SD and analyzed using two-tailed t-test (**A**,** C**), one-way ANOVA followed by Tukey’s test (**B**). * *p* < 0.05, **p < 0.01, ****p < 0.0001

Lipofuscin accumulation, a marker of non-degradable lipid and protein aggregates in the cytoplasm of stressed or damaged cells, is one of the hallmarks of cellular senescence [49]. Using SenTraGor (STG, a reagent for detection of lipofuscin) [50] staining in postmortem human brain slices (mid-frontal lobe region) from ROS, we observed higher STG intensity in GFAP-positive astrocytes in AD participants compared to NCI participants with the APOE3/4 genotype (Fig. 2A, the STG/GFAP staining test is shown in Supplementary Fig. 2A), corroborating the increased astrocyte senescence markers in AD observed in the transcriptomic data.

To determine whether ABCA1 expression differed by AD and APOE4, ABCA1 protein levels were examined in postmortem human brain tissue of individuals with NCI and AD dementia, grouped by APOE4 status. Given that ABCA1 is predominantly localized in cell membranes [51], cytosolic proteins were removed (Fig. 2B, Supplementary Fig. 2B). Total membrane ABCA1 levels were higher in APOE3/3 and APOE3/4 carriers with AD dementia than in APOE3/3 and APOE3/4 carriers in the NCI group (Fig. 2B; full blots are shown in Supplementary Fig. 2C). However, no differences in ABCA1 levels were detected according to APOE genotype (Fig. 2B). Because an increase in lysosomal ABCA1 can import cholesterol to lysosomes to drive mTOR activation [7], we fractionated postmortem tissues to enrich lysosomes. ABCA1 was more abundant in lysosome-enriched fractions in the AD group than in the NCI group within the APOE3/4 genotype but not in the APOE3/3 genotype (Fig. 2C, full blots are shown in Supplementary Fig. 3), supporting that more ABCA1 was trapped in lysosomes in APOE4 AD. We also observed a positive correlation between phosphorylated mTOR levels and ABCA1 (Fig. 2D; R^2^ = 0.59; *p* < 0.0001), implying that lysosomal ABCA1 contributed to mTOR activation in AD.

### Proteomic experiments identify caveolin-1 as a mechanism for ABCA1 trafficking to lysosomes following ApoE4 treatment

**Fig. 3:**
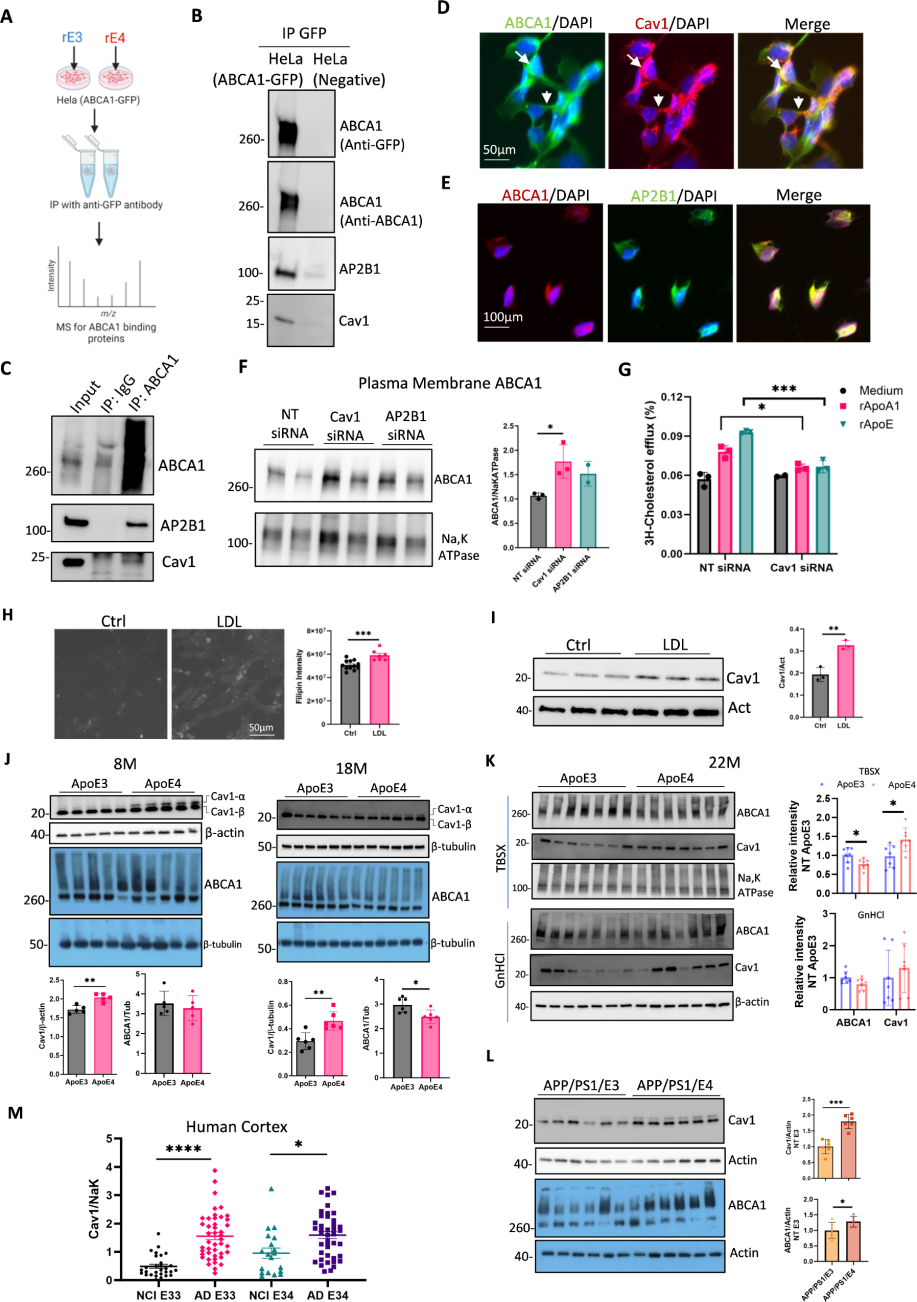
Caveolin-1 regulates ABCA1 trafficking in cells and its expression is increased in APOE4 and AD biospecimens. (**A**) Schematic overview of the workflow for purification of the ABCA1 complex. (**B**) Co-immunoprecipitation of ABCA1 in ABCA1-GFP expressing and wild-type HeLa cells. (**C**) Co-immunoprecipitation in lysates of immortalized astrocytes using an ABCA1 antibody or species-matched IgG. ABCA1, Caveolin-1 and AP2B1 were detected by immunoprecipitation. (**D**) Co-staining for ABCA1 and caveolin-1 in immortalized astrocytes. Arrow indicates the cytoplasm, and arrowhead indicates the plasma membrane. (**E**) Co-staining for ABCA1 and AP2B1 in immortalized astrocytes. (**F**) Mouse primary astrocytes were transfected with non-target (NT), caveolin-1, and AP2B1 siRNA for 48 h. Plasma membrane protein was enriched by biotin agarose beads and the ABCA1 protein levels were detected by WB. Quantification was performed from two–three independent experiments. (**G**) Immortalized astrocytes were transfected with non-target or caveolin-1 siRNA for 24 h, followed by labeling with 3 H-cholesterol for 18 h. Cholesterol efflux was measured after treatment with recombinant ApoA1 or ApoE for 4 h (*n* = 3 biological replicates). (**H**) Filipin staining of immortalized astrocytes loaded with or without low-density lipoprotein (LDL) (10 µg/mL) for 24 h. (*n* = 7–12 random areas from two cultured wells). (**I**) Caveolin-1 protein levels in immortalized astrocytes loaded with or without LDL (10 µg/mL) for 24 h were detected by WB (*n* = 3 biological replicates). (**J**) Protein levels of caveolin-1 and ABCA1 in the cortex of 8-months-old and 18-months-old APOE3 and APOE4-TR mice (*n* = 5–6 mice for each genotype, mixed gender). (**K**) For 22-months-old APOE3 and APOE4-TR mice, the cortex was sequentially homogenate with TBS, TBSX and GnHCl. ABCA1 and caveolin-1 protein levels in TBSX-soluble (membrane enriched) and GnHCl-soluble (aggregated protein enriched) fractions were measured by WB. (*n* = 7 mice for each genotype, both sexes). **(L)** Protein levels of caveolin-1 and ABCA1 in the cortex of 6-months-old APP/PS1/APOE3 and APP/PS1/APOE4 mice were detected by WB (*n* = 6 mice of each genotype, both sexes). (**M**) Total membrane caveolin-1 protein levels in human postmortem mid-frontal lobe tissues from reactive oxygen species (ROS) were detected by WB. Quantification of the relative intensity of caveolin-1 normalized to the plasma membrane marker Na, K-ATPase (NCI APOE3/3, *n* = 29; AD APOE3/3, *n* = 44; NCI APOE3/4, *n* = 19; AD APOE3/4, *n* = 42). Data are represented as mean ± SD and analyzed using two-tailed t-test (**F**,** G**,** H**,** I**,** J**,** K and L**) or one-way ANOVA followed by Tukey’s test (**M**). * *p* < 0.05, ** *p* < 0.01, *** *p* < 0.001, **** *p* < 0.0001

We previously reported that APOE4 leads to more ABCA1 in astrocyte lysosomes than APOE3 [31]. Here, we confirmed these findings in lysosome-enriched fractions of human brain tissue from patients with APOE4 AD. To explore the underlying mechanism, we treated ABCA1-GFP overexpressing HeLa cells with recombinant ApoE3 (rE3) or ApoE4 (rE4). rE4 treatment resulted in lower total ABCA1 protein levels than rE3 treatment, indicating greater ABCA1 degradation (Supplementary Fig. 4A). ABCA1-binding complexes were enriched for proteomic analysis (Fig. 3A and Supplementary Fig. 4B). Proteomic analysis revealed 768 shared proteins between rE3 and rE4 treatments, with 314 proteins unique to rE4 (Supplementary Fig. 4C). Subsequent experiments focused on the endocytic pathway given its potential role in protein degradation [52, 53]. Nine of these proteins (Supplementary Table 4) showed higher expression in the rE4 treatment group than in the rE3 group (Supplementary Fig. 4D). Internalization of surface ABCA1 has been reported to be regulated by multiple endocytosis mechanisms, including clathrin-mediated, caveolin-mediated, and GTPase ADP ribosylation factor 6 (ARF6) mediated pathways [54]. Caveolin-1 (Cav-1), linked to caveolae-dependent endocytosis, and the adaptor-related protein complex 2 subunit beta 1 (AP2B1), linked to clathrin-dependent endocytosis [52], were further validation by co-immunoprecipitation. Both Caveolin-1 and AP2B1 were detected in complexes with ABCA1, suggesting that they are involved in ABCA1 trafficking (Fig. 3B and C). ABCA1, caveolin-1, and AP2B1 colocalized in both the membrane and intracellular compartments of astrocytes (Fig. 3D, and E, respectively). Reducing caveolin-1, but not, AP2B1 expression by siRNA increased plasma membrane ABCA1 levels in mouse primary astrocytes (Fig. 3F, Supplementary Fig. 4E), supporting that caveolae-mediated endocytosis regulated ABCA1 degradation. Surprisingly, reducing caveolin-1 expression significantly decreased ABCA1-mediated cholesterol efflux (Fig. 3G) despite an increase in total ABCA1 protein levels (Supplementary Fig. 4F). Furthermore, overexpression of caveolin-1 in astrocytes enhanced cholesterol efflux (Supplementary Fig. 4G). These results indicate that caveolin-1 not only traffics ABCA1 to the lysosome but also plays a critical role in promoting ABCA1-mediated cholesterol efflux, possibly by enriching the plasma membrane with cholesterol or facilitating the recycling of ABCA1 between membrane and intracellular pools.

### The association of APOE4 with increased caveolin-1 expression indicates cholesterol accumulation

Caveolae, plasma membrane invaginations, and increased cellular cholesterol accumulation [55] were measured using filipin staining in an immortalized mouse astrocyte cell line. Cholesterol loading, by adding LDL, led to increased caveolin-1 expression (Fig. 3H and I) and showed a dose-dependent increase in baby hamster kidney (BHK) cells (Supplementary Fig. 4H). Since APOE4 is known to induce greater cholesterol accumulation than APOE3 [56], we examined whether APOE4 was associated with higher caveolin-1 protein levels in the cortical tissues of APOE4-and APOE3-TR mice at 8 and 18 months of age. Higher caveolin-1 protein levels, including the α and β isoforms, were found in APOE4-TR mice than in APOE3-TR mice at 8 months of age (Fig. 3J). At 18 months of age, ABCA1 protein levels were lower and caveolin-1 protein levels were higher in the cortical tissues of APOE4-TR mice than in APOE3-TR mice (Fig. 3J).

Mouse cortices were fractionated using TBSX and guanidine HCl to isolate soluble membrane and insoluble aggregated proteins (Supplementary Fig. 5A). Further analysis showed that in cortices from 22-month-old APOE3-TR and APOE4-TR mice, caveolin-1 protein levels in the membrane protein-enriched fraction were significantly higher in APOE4-TR mice than in APOE3-TR mice, while membrane ABCA1 levels were lower in APOE4-TR mice (Fig. 3K). No differences in caveolin-1 and ABCA1 levels were observed in the insoluble aggregated protein fraction between the APOE3 and APOE4 TR mice (Fig. 3K). Interestingly, higher caveolin-1 and ABCA1 protein levels were found in the cortex of APP/PS1/APOE4 mice than in that of APP/PS1/APOE3 mice (Fig. 3L). In human postmortem middle frontal lobe tissues, caveolin-1 protein levels were higher in both APOE3/3 and APOE3/4 carriers with AD dementia than in the NCI group, although there was no difference between the APOE3/3 and APOE3/4 genotypes in either group (Fig. 3M; full blots are shown in Supplementary Fig. 2C). These results suggest that APOE4 is associated with the accumulation of cellular cholesterol, inducing caveolin-1 expression to compensate for reduced cholesterol efflux. However, cholesterol accumulation also increases caveolae-dependent ABCA1 endocytosis, trapping ABCA1 in lysosomes.

To identify the main cell type in which ABCA1 and caveolin-1 interact, we isolated microglia, astrocytes and axon-enriched fraction from the brains of adult mice (Supplementary Fig. 5B). Astrocytes were enriched in the CD11b^-^ fraction (Supplementary Fig. 5C). ABCA1 was mainly expressed in astrocytes and axon-enriched fractions, and lower ABCA1 expression was detected in microglial cells (Supplementary Fig. 5D). Caveolin-1 was mainly expressed in astrocytes and microglia but not in axon-enriched fractions (Supplementary Fig. 5E). These results indicated that the interaction between ABCA1 and caveolin-1 largely occurs in astrocytes.

### Lysosomal ABCA1 mediates mTOR activation and senescence triggered by cholesterol accumulation

**Fig. 4:**
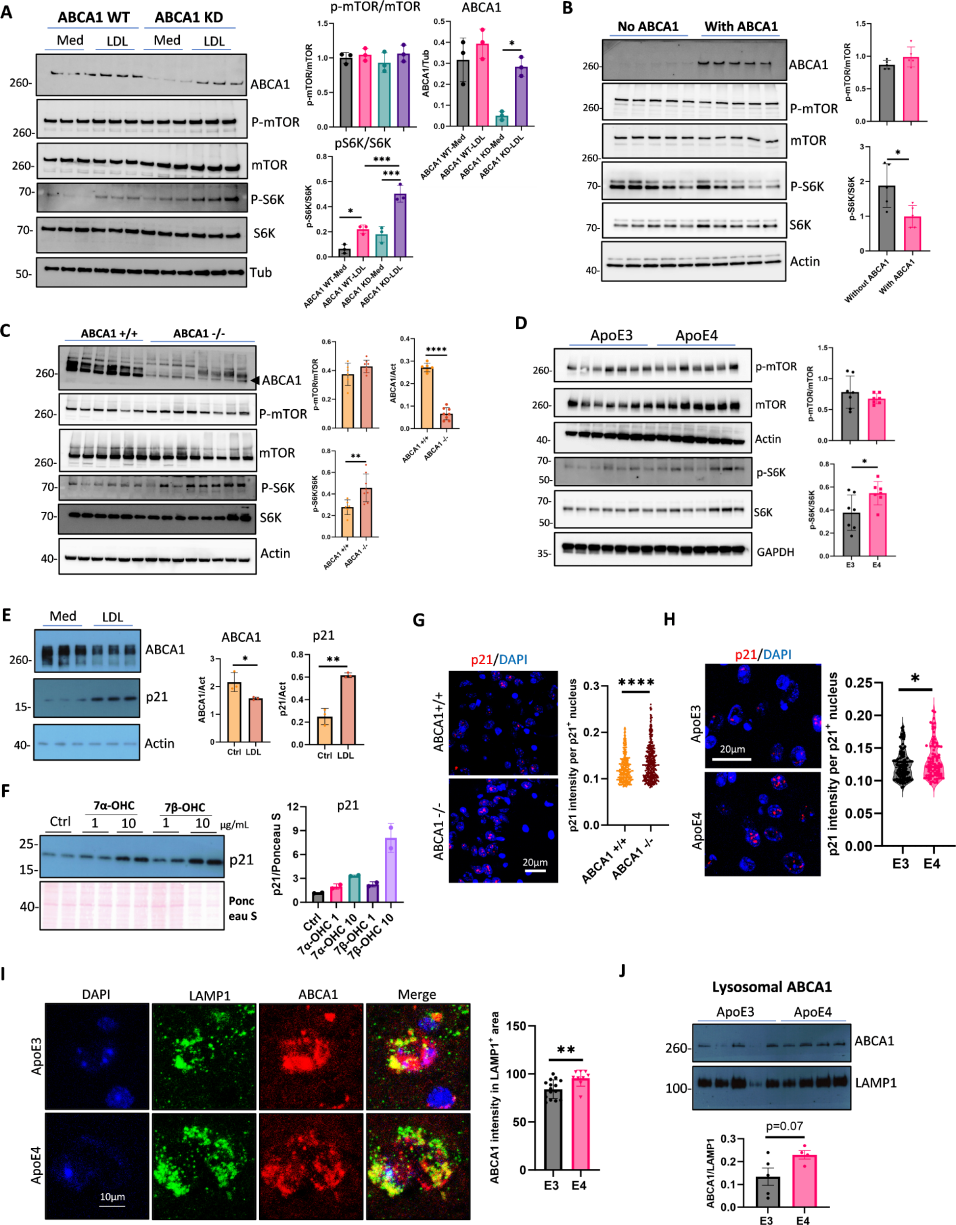
ABCA1 mediates mTORC1 activation and cellular senescence. (**A**) ABCA1 knockdown astrocytes were treated with MβCD (5 mg/mL) for 2 h, followed by incubation with control medium (Med) or LDL (100 µg/mL) for 2 h. ABCA1, total and phosphorylated mTOR, and S6K were detected by WB (*n* = 3 biological replicates). (**B**) BHK cells were treated with mifepristone (0.1 nM) for 16 h to induce ABCA1 expression, followed by treatment with LDL (100 µg/mL) for 24 h. ABCA1, total and phosphorylated mTOR, and S6K were detected by WB (*n* = 5 biological replicates). (**C**) The cortex of 10-month-old ABCA1 brain conditional knockout mice was homogenized in RIPA buffer. ABCA1, total and phosphorylated mTOR, and S6K were detected by WB. (*n* = 6 for ABCA1 WT mice, *n* = 8 for ABCA1 knockout mice of both sexes). (**D**) Phosphorylated and total mTOR, and S6K in the cortex of 22-month-old APOE3 and APOE4-TR mice were detected by WB (*n* = 7 for each genotype, both sexes). (**E**) Astrocytes were loaded with medium (control) or LDL (100 µg/mL) for 24 h. ABCA1 and p21were detected by WB (*n* = 3 biological replicates). (**F**) Astrocytes were loaded with medium (Ctrl), 7α-OHC (7α-hydroxycholesterol) or 7β-OHC (7β-hydroxycholesterol) for 24 h. p21 was detected by WB (*n* = 2 biological replicates). (**G**) Representative images of p21 staining by immunofluorescence in the ABCA1 WT and ABCA1 brain conditional knockout mouse brain slides. (*n* > 300 nuclei from 6 mice in each group). (**H**) Representative images of p21 staining by immunofluorescence in the brain of APOE3 and APOE4-TR mice (*n* > 90 nuclei from 5 mice in each group). (**I**) Representative images of LAMP1 and ABCA1 staining by immunofluorescence in the APOE3 and APOE4-TR mouse (14-month-old) brain slides (*n* = 9–15 LAMP1^+^ ROIs from 3 mice in each group). (**J**) Lysosomes were isolated from brains of ApoE3 and ApoE4-TR mice (14-month-old). ABCA1 protein levels in lysosome were measured by WB. (*n* = 4–5 each group, mixed gender). Data are represented as the mean ± SD and analyzed using a two-tailed t-test (**B-J**) or one-way ANOVA followed by Tukey’s test (**A**). * *p* < 0.05, ** *p* < 0.01, *** *p* < 0.001, **** *p* < 0.0001

Previous studies in fibroblast cell models have shown that cholesterol accumulation in lysosomes activates mTORC1 via ABCA1, inducing cellular senescence and SASP [57–59]. To examine the role of lysosomal ABCA1 in mTORC1 activation in brain cells, an ABCA1 knockdown astrocyte cell line was created using CRISPR-cas9. When cholesterol was loaded into these cells for 2 h, a significant increase in p-S6K/S6K levels, a key marker of mTORC1 activation, was observed in cells with reduced ABCA1 expression (Fig. 4A). Conversely, ABCA1 overexpression in BHK cells reduced cholesterol-induced mTORC1 activation (Fig. 4B). Similarly, higher p-S6K/S6K levels were found in the cortex of 10-month-old conditional brain ABCA1 knockout mice (ABCA1^fl/fl^ mice crossed with Nestin Cre mice, Fig. 4C) and in APOE4-TR mice compared to controls (Fig. 4D). These findings suggest that the induction ABCA1 activity decreases mTOR activation in models of cholesterol accumulation.

To test the effect of prolonged cholesterol exposure on senescence, astrocytes were treated with LDL for 24 h. This resulted in an increase in the nuclear senescence marker p21 together with reduced ABCA1 expression (Fig. 4E), corroborating that loss of ABCA1 function is associated with cholesterol-induced senescence. Higher concentrations of both 7α- and 7β-OHC increased nuclear senescence marker p21 after 24 h of treatment (Fig. 4F). In agreement, ABCA1 brain knockout mice showed higher p21 intensity in the brain than ABCA1 WT mice (Fig. 4G, p21 staining test is shown in Supplementary Fig. 6A). Consistent with this model, higher p21 intensity was also found in the brains of APOE4-TR mice than in APOE3-TR mice (Fig. 4H). To indirectly assess lysosomal ABCA1 localization in APOE4-TR brains, tissue slides were double stained with LAMP1 and ABCA1 (Supplementary Fig. 6B). We observed greater colocalization of LAMP1 with ABCA1 in APOE4-TR mice than in APOE3-TR mice (Fig. 4I). Lysosomes isolated from mouse brains showed higher ABCA1 protein levels in the lysosomal fractions of APOE4-TR mice (Fig. 4J). Taken together, these results suggest that with cholesterol loading, reduced ABCA1 cholesterol transport activity is associated with increased markers of cellular senescence. APOE4 exhibits more markers of brain cellular senescence than APOE3, which can be explained by more ABCA1 being trapped in lysosomes and less ABCA1 participating in cholesterol transport. These findings suggest that modulating ABCA1 recycling from lysosomes could be a promising strategy to mitigate cholesterol-induced mTOR activation and senescence, especially in APOE4 carriers. This insight opens up potential avenues for therapeutic interventions aimed at restoring cholesterol homeostasis and reducing cellular senescence.

### Reducing oxysterols rescues lysosomal ABCA1 trapping and promotes endosome-lysosome recycling

**Fig. 5:**
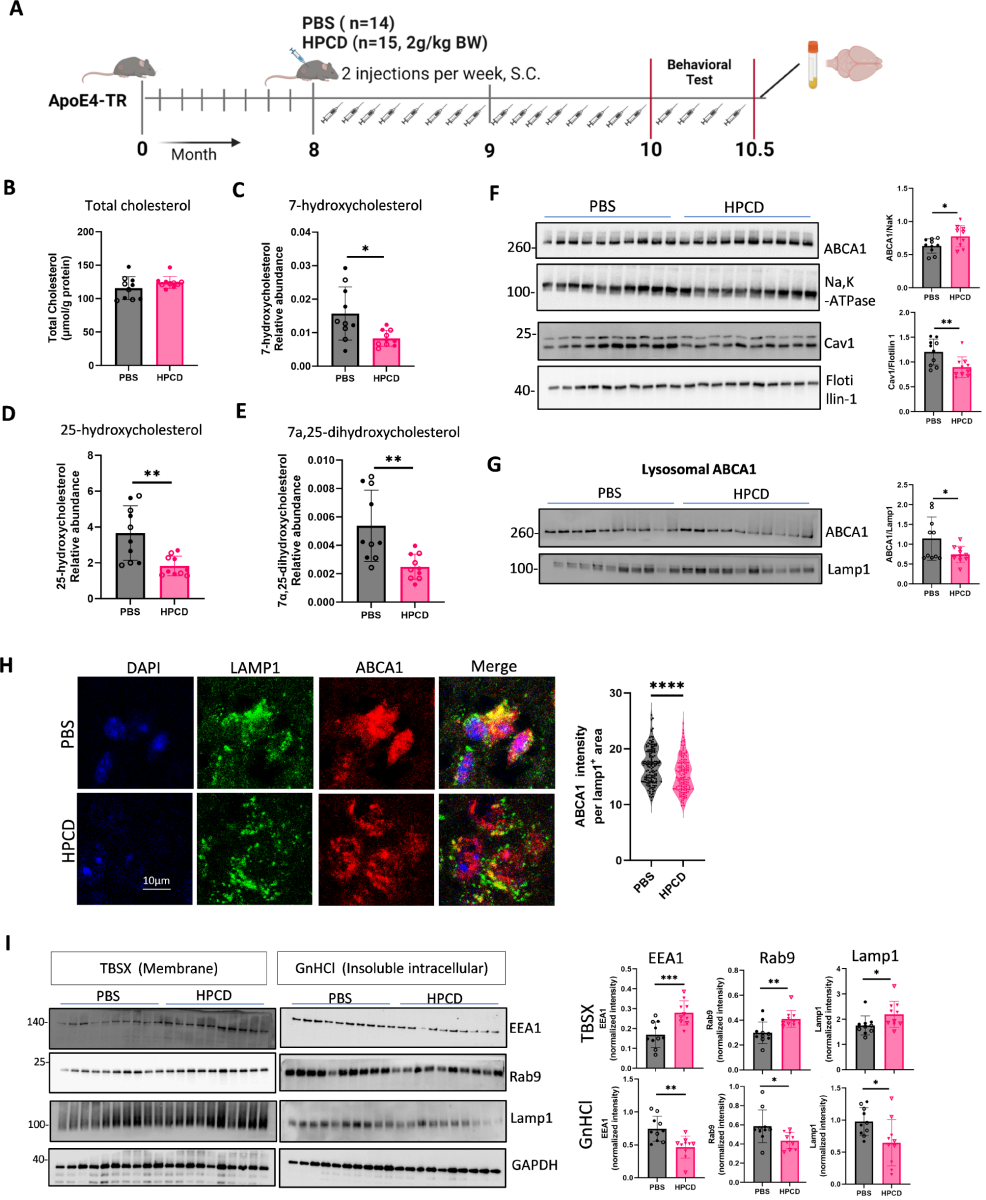
Reduction of oxysterols by HPCD increases ABCA1 and endo-lysosome recycling in APOE4-TR mice. (**A**) Study design and timeline for mouse experiments. APOE4-TR mice were injected with (2-Hydroxypropyl)-β-cyclodextrin (HPCD) for 2 months, followed by behavioral and pathological tests (*n* = 14, 5 females and 9 males for PBS group; *n* = 15, 5 females and 10 males for the HPCD group). (**B**) Total cholesterol levels in mouse brain homogenates. (**C-E**) Oxysterols including 7β-hydroxycholesterol (**C**), 25-hydroxycholesterol (**D**) and 7α,25-dihydroxycholesterol (**E**) levels in mouse brain were measured by mass spectrometry. (**F**) Total membrane protein levels of ABCA1 and caveolin-1 in the mouse cortex detected by WB. (**G**) Lysosomes were isolated from mouse brains. ABCA1 protein levels in lysosomes were detected by WB. (**H**) Representative confocal images of LAMP1 and ABCA1 staining in mouse brain sections. Quantification of ABCA1 intensity in LAMP1^+^ area. (*n* = 150 LAMP1^+^ ROI from 5 mice in each group; A.U.). (**I**) Protein levels of EEA1 (early endosome marker), Rab9 (late endosome marker), and LAMP1 (lysosome marker) in the membrane fraction (TBSX) and the intracellular fraction (GnHCl) were detected by WB. (*n* = 10, five females and five males in each group for B-G, and I). Data are represented as mean ± SD and analyzed using a two-tailed t-test. * *p* < 0.05; ** *p* < 0.01, *** *p* < 0.001, **** *p* < 0.0001. Females are indicated with empty dots and males are indicated with filled dots for all data plots

To test whether reducing cholesterol metabolites in ApoE4 rescues ABCA1 trapping and senescence, we treated APOE4-TR mice with hydroxypropyl-β-cyclodextrin (HPCD) for 2 months starting at 8 months of age (*n = 14* PBS- and *n* = 15 HPCD-treated mice), followed by behavioral and biochemical testing (Fig. 5A). Body weight was not affected by HPCD treatment (Supplementary Fig. 7A). Total cholesterol levels in the brain (Fig. 5B) or plasma (Supplementary Fig. 7B) were also no different between HPCD and PBS groups. However, levels of oxysterols (7-hydroxycholesterol, 25-hydroxycholesterol, and 7α,25-dihydroxycholesterol) were reduced in HPCD-treated mice compared to controls (Fig. 5C-E). Brain tissue fractionation showed a significant decrease in total membrane caveolin-1 levels in HPCD treated-APOE4-TR mice, along with enhanced ABCA1 recycling, as indicated by increased membrane ABCA1 levels (Fig. 5F). Lysosomal ABCA1 levels were lower in the HPCD treatment group, as verified by lysosome isolation and immunostaining (Fig. 5G and H; validation of lysosome isolation is shown in Supplementary Fig. 7C). Treatment with HPCD was associated with more efficient cholesterol efflux, as evidenced by increased trends of small HDL particle formation in cerebrospinal fluid (CSF) and plasma, although these changes did not reach statistical significance due to the small sample size (Supplementary Fig. 7D-F).

Further analysis revealed that HPCD treatment led to more soluble and less aggregated levels of key endolysosomal markers, including EEA1 (early endosomes), Rab9 (late endosomes), and LAMP1 (lysosomes), in the membrane fraction, an indicator of restored endosome-lysosomal recycling (Fig. 5I).

### Reduction of oxysterols by HPCD decreases senescence and neuroinflammation markers, and rescues cognitive deficit in APOE4-TR mice

**Fig. 6:**
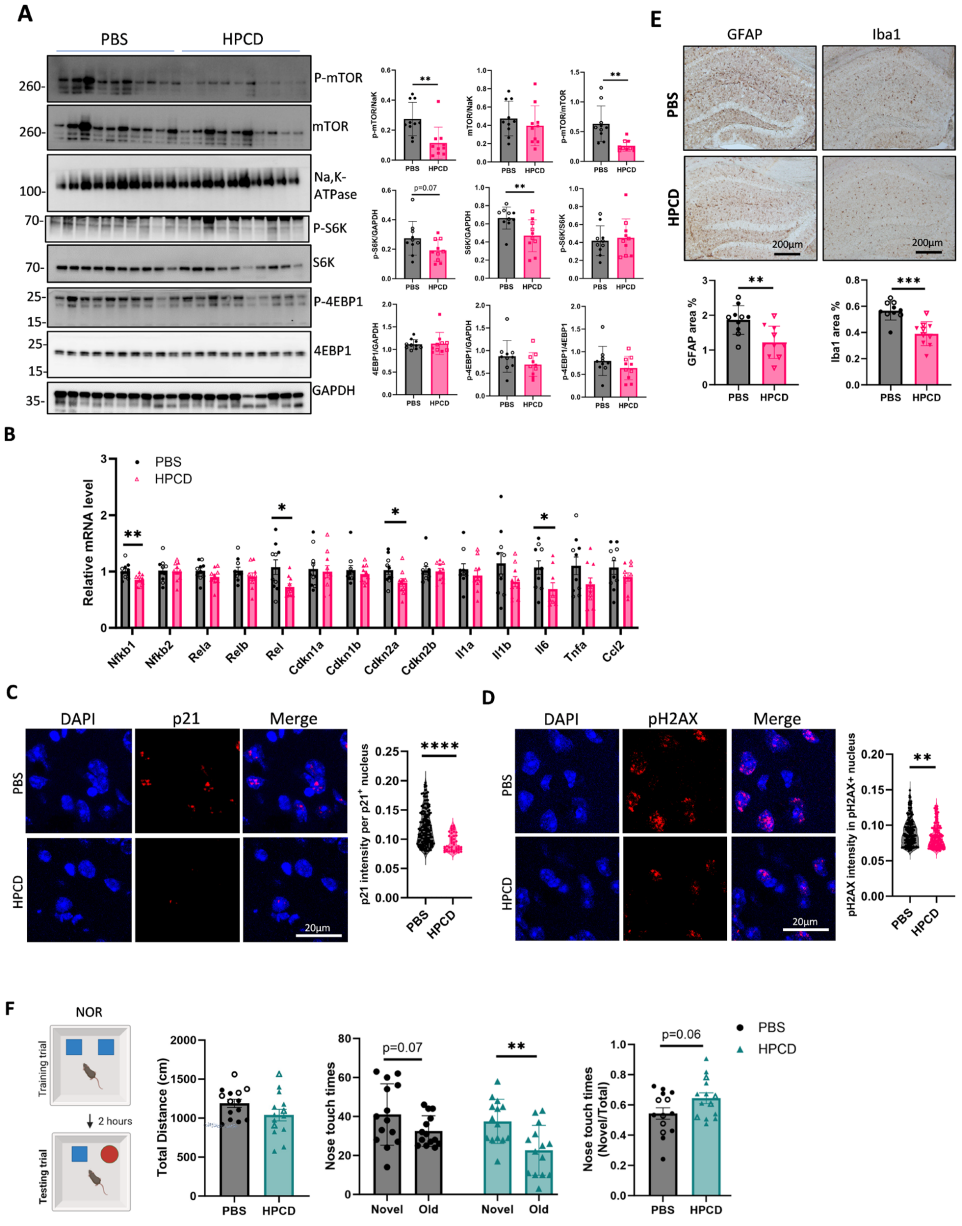
Reduction of oxysterol by HPCD reduces mTORC1 activation, senescence and neuroinflammation in APOE4-TR mice. (**A**) Phosphorylated and total mTOR, S6K, and 4EBP1 levels in the cortex of APOE4 mice treated with or without HPCD were detected by western blotting (*n* = 10 per group). (**B**) The mRNA levels of NF-κB, cellular senescence markers, and cytokines in the mouse cortex were determined using qPCR (*n* = 10 per group). (**C**) Representative images of p21 staining by immunofluorescence in the brain slide of APOE4-TR mice treated with PBS or HPCD (*n* > 40 nuclei from 5 mice in each group). (**D**) Representative images of pH2A.X staining by immunofluorescence in the brain slide of APOE4-TR mice treated with PBS or HPCD (*n* > 160 nuclei from 5 mice in each group). (**E**) Representative images and quantification of activated astrocytes and microglia stained with GFAP and Iba-1 antibodies, respectively, in the hippocampi of mice treated with PBS or HPCD (*n* = 10 per group). (**F**) Recognition ability of mice treated with PBS or HPCD was assessed using the novel objective recognition (NOR) test. The total distance moved during the test trial, nose touch times for both test trial objectives, and the ratio of novel to total nose touch times in the testing trial were analyzed using the EthoVision XT software (*n* = 14 per group). Data are represented as mean ± SD and analyzed using a two-tailed t-test. * *p* < 0.05; ** *p* < 0.01, *** *p* < 0.001, **** *p* < 0.0001. Females are indicated with empty dots and males are indicated with filled dots

Decreasing oxysterol levels with HPCD treatment reduced the expression of mTORC1 activation markers (Fig. 6A), lowered NF-kB members (Nfb1, Rela, Rel), and some senescence and SASP markers (Cdkn2a (p16) and Il-6 mRNA levels, Fig. 6B), and decreased p21 and H2A.X intensity (Fig. 6C and D) in mouse brains. HPCD also diminished the activated astrocytes and microglia (Fig. 6E), and several inflammatory cytokines (Supplementary Fig. 8A), but increased synaptophysin, PSD95, and vGLuT1 levels (Supplementary Fig. 8B and 8 C). In the behavioral tests, HPCD-treated mice showed improved performance in the novel object recognition test (Fig. 6F). To validate these findings in human cells, we cultured human induced pluripotent stem cell (hiPSC)-derived astrocytes with APOE4/4 genotype (Supplementary Fig. 9A). When these human iPSc-derived astrocytes were treated with methyl-β-cyclodextrin (MβCD) after LDL loading, cellular cholesterol accumulation (Supplementary Fig. 9B) and IL-1β and CCL2 mRNA levels were decreased (Supplementary Fig. 9C).

## Discussion

This study uncovers unique markers of cellular senescence signatures in astrocytes, excitatory neurons, microglia, and other immune cells that correlate with more severe AD neuropathology, particularly in patients carrying the APOE4 allele. Using a relatively large and well-phenotyped human brain cohort, we verified that the severity of cellular senescence escalates with increased Braak stages and neuritic plaques. Across different cell types, ABCA1 expression was strongly correlated with certain markers of cellular senescence and the accumulation of oxysterols, rather than total cholesterol levels. Using cellular and animal models, our findings implicate the accumulation of oxysterols in lysosomal dysfunction, reduction of ABCA1 recycling, reduction of cholesterol efflux activity, and sequestration of ABCA1 in lysosomes as drivers of cellular senescence phenotypes in the APOE4 AD brain.

Although it is unclear whether certain features of cellular senescence cause or result from neurodegeneration [2], growing evidence confirms their presence in multiple cell types, including neurons, astrocytes, microglia, endothelial cells, and oligodendrocyte precursor cells, in AD mouse models and, importantly, in AD human brains [5, 43,13, 60–62]. Our analysis is consistent with recent single-cell transcriptomic studies that showed that excitatory neurons are the most senescent cells in the human brain [10]. Amyloid beta (Aβ) and tau accumulation are major inducers of cellular senescence in AD [11, 62], and the removal of senescent cells has been shown to alleviate AD pathology and enhance memory in AD mouse models [12, 13]. Whether cells that do not display senescent phenotypes, such as inhibitory neurons, are more vulnerable to cell death remains unknown.

Dysregulated cholesterol homeostasis is a key contributor to inflammation and senescence in AD. Previous studies have linked a high-fat diet and specific cholesterol metabolites to increased inflammatory signaling in low-density lipoprotein receptor-knockout mice [63], in the kidneys of C57BL/6J mice [64], and in in vitro human lung cells [22]. In the context of AD, cholesterol and lipid accumulation has been reported in cellular and mouse models of Aβ [65, 66], tau [67, 68], and APOE4 [47, 69–71]. A recent study identified more senescent neurons in the hippocampus of old ApoE4-TR mice than in ApoE3-TR mice because of dysregulation of ATP and acetyl coenzyme A [18], which are substrates for cholesterol synthesis [72]. Our research suggests that in APOE4, cholesterol shift to lysosomes contributes to mTORC1 activation and the pro-inflammatory senescence-associated secretory phenotype (SASP) [7, 73]. ABCA1 plays a pivotal role in this process by importing cholesterol into lysosomes and potentially leading to lysosomal dysfunction [73]. ABCA1 expression in lysosomal compartments has been implicated in the accumulation of lipid droplets [74]. Caveolin-1, a critical regulator of ABCA1 degradation and cholesterol homeostasis, appears to mediate the sequestration of ABCA1 in lysosomes, further disrupting cholesterol transport and contributing to cellular senescence [75]. Importantly, caveolin-1 can act as a cholesterol sensor and regulate cellular cholesterol homeostasis [75, 76]. Cholesterol accumulation in APOE4 and AD cells was reflected by increased caveolin-1 expression.

Our results suggest that restoring cholesterol homeostasis presents a viable therapeutic strategy to mitigate the effects of AD. Lowering cholesterol by cyclodextrin increases axonal myelination and improves learning and memory in APOE4-TR mice [71]. In AD mouse models, the reduction of cholesterol in astrocytes robustly reduces amyloid and tau burden [77]. Clearing out of the cholesterol burden promotes the clearance of defective mitochondria via autophagy in cells and ameliorates brain inflammation and neurodegeneration in the APOE4/Tau mouse model [67, 78]. Our results implicate oxysterol metabolism as a druggable axis for AD treatment, compared to total cholesterol. Unfortunately, to date, no clinical intervention focusing on modulating cholesterol metabolism has been successful in patients with AD. Examples of these interventions include rosiglitazone [79], pioglitazone (PPARγ agonists) [80], and bexarotene (RXR agonist) [81], which are ineffective in APOE4 carriers with AD. Our findings indicate that interventions that induce ABCA1 without accounting for endolysosomal dysfunction may inadvertently accelerate ABCA1 lysosomal sequestration. This concept highlights the importance of drugs or therapeutics that target endolysosomal functions and ABCA1 recycling.

Our study had a few limitations. We recognize that APOE-TR mice are not a good model of cellular senescence, but our findings support that they can be useful in dissecting the effects of aging and APOE4 on certain markers of cellular senescence, such as p21. We did not identify the mechanisms by which APOE4 induced oxysterols affect lysosomal recycling. Prior studies have suggested that targeting NHE6, the primary proton leak channel in the early endosome, can reverse the ApoE4-induced recycling block of several receptors [82]. It is plausible that oxysterols directly or indirectly affect endolysosomal pH and recycling. With the relatively small sample size of CSF obtained from mouse studies, the changes in HDL particles after treatment did not reach statistical significance. Finally, caveolin-1 expression does not appear to be a good drug target for restoring ABCA1 recycling, as reducing its expression was associated with lower cholesterol efflux.

**Fig. 7:**
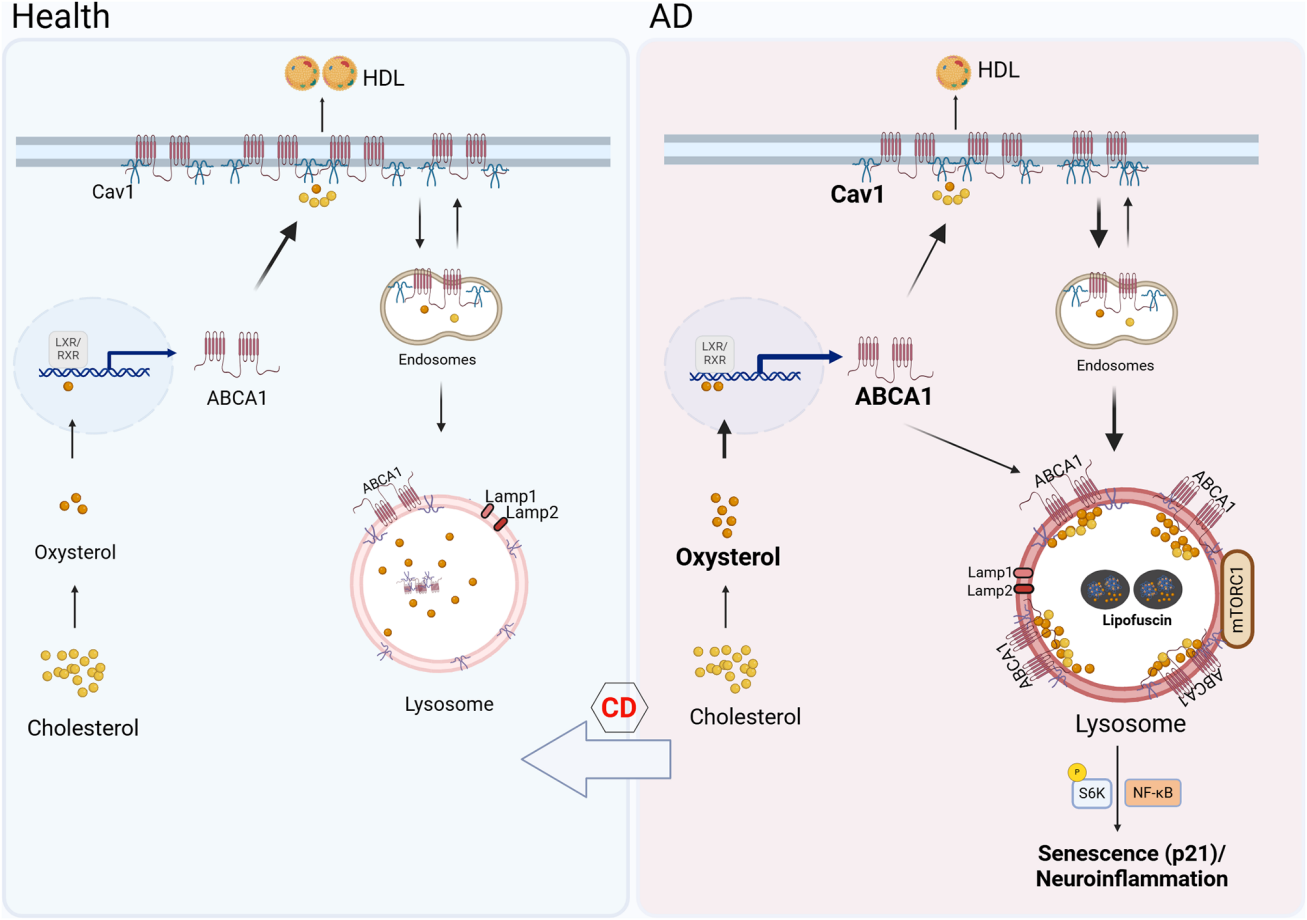
Oxysterol accumulation promotes cellular senescence and neuroinflammation in APOE4 and AD through the aggregation of ABCA1 in lysosomes. Compared to healthy cells, a higher oxysterol burden in AD, particularly in APOE4 carriers, leads to increased ABCA1 and caveolin-1 expression, and lysosomal dysfunction marked by increased lipofuscin staining. This sequestration reduces ABCA1 endosome-lysosome recycling, and exacerbates cholesterol and lipid accumulation within lysosomes, activating mTORC1, and contributing to cellular senescence and neuroinflammation. Treatment with cyclodextrin mitigates these effects by lowering oxysterols. Illumination is created by BioRender. CD: Cyclodextrin

## Conclusions

In conclusion, we demonstrated that oxysterol accumulation in APOE4 and AD promotes greater expression of ABCA1 and caveolin-1, which endocytoses and traps ABCA1 in lysosomes and induces a dysfunctional lysosomal state in which ABCA1 fails to recycle back to the plasma membrane. This process activates the mTORC1 pathway and induces cellular senescence and neuroinflammation. The reduction in oxysterols by cyclodextrin decreased lysosomal ABCA1, accelerated endolysosomal recycling, increased the efflux of cholesterol to HDL, and reduced mTORC1 activation, senescence, and neuroinflammation (Fig. 7). Future research should explore the detailed mechanisms of endolysosomal recycling in AD, as well as more potent and specific compounds that modulate cholesterol homeostasis. This could lead to the development of more effective treatments that can restore cholesterol balance, reduce cellular senescence, and ultimately mitigate AD progression, especially in APOE4 carriers.

## Electronic supplementary material

Below is the link to the electronic supplementary material.

**Supplementary Material 1: Figure 1.** Single-nucleus RNA (sn-RNA) sequencing analysis of the association between senescence-associated secretory phenotype (SASP) (A) and mTOR expression (B) in different brain cells with factors including AD pathology, sex, APOE4 genotype, and ABCA1 expression (Related to Fig. 1).

**Supplementary Material 2: Figure 2.** Validation of immunofluorescent staining and whole blotting of western blots. (A) Validation of STG and GFAP staining. The negative control refers to omitting the primary antibody. (B) Frozen human postmortem mid-frontal lobe tissues were homogenized with different buffers and ABCA1 expression was detected by WB. Beta-actin and Na +/K + ATPase were used as markers for different fractions. (C) Total membrane ABCA1 and caveolin-1 protein levels in human postmortem mid-frontal lobe tissues were detected by WB. (NCI E3/3, n=33; NCI E3/4, n=19; AD E3/3, n=44; AD E3/4, n=42. One sample labelled with a star was excluded as it had MCI) (Related to Fig. 2).

**Supplementary Material 3: Figure 3.** The full blot of ABCA1 detection in lysosome membrane enriched fraction in human brain homogenate (immunoprecipitation with anti-LAMP2 antibody) (Related to Fig. 2C).

**Supplementary Material 4: Figure 4.** Caveolin-1 regulates ABCA1 degradation. (A) HeLa cells expressing ABCA1-GFP were treated with recombinant ApoE3 or ApoE4 (0.2μM) for different hours. Cells were lysed with RIPA buffer and total ABCA1 levels were detected by WB using an anti-GFP antibody. The lower panel shows the densitometric quantification of the blots shown in the upper panel. ABCA1 was normalized to β-actin and then normalized to rApoE3 at the 0 h point. (B) Immunoprecipitation was performed with GFP-trap agarose in the lysate of HeLa cells or HeLa (ABCA1-GFP) cells treated with or without recombinant APOE3 (rE3) or APOE4 (rE4) for 4 h. Elution was detected using sodium dodecyl sulfate-polyacrylamide gel electrophoresis with silver staining. (C) 1115 and 1082 ABCA1 binding targets were detected in the recombinant APOE3 (rE3) and recombinant APOE4 (rE4) treatment groups, respectively, whereas 768 targets were found in both rE3 and rE4 groups. The top 14 candidates of the enriched KEGG pathways were identified from these 768 common targets (FDR < 0.05). (D) Enrichment levels (rE4 vs. rE3) of targets in the endocytic pathway. NSAF was used to calculate the fold change of each target. See the methods for calculating the NSFA. (E) Primary astrocytes were transfected with 15nM of non-target (NT), Caveolin-1 and AP2B1 siRNA for 48 h. The total protein levels of caveolin-1 and AP2B1 were detected by WB after siRNA transfection. (F) Immortalized astrocytes were transfected with 20nM of non-target (NT) or caveolin-1 siRNA for 48 h. The total protein levels of ABCA1 and caveolin-1 were detected by WB after siRNA transfection (n=3 biological replicates). (G) Caveolin-1 is overexpressed in immortalized astrocytes for 24 h, followed by labeling with 3H-cholesterol for 18 h. Cholesterol efflux was measured after treatment with the CS6253 peptides for 4 h (n=3 biological replicates). The right panel shows ABCA1 and caveolin-1 protein levels after plasmid transfection. (H) BHK cells were treated with LDL for 24 h, and caveolin-1 protein levels were detected by WB (n=3 biological replicates). Data are represented as mean± SD and were analyzed using unpaired t-test (E, F), one-way ANOVA (H), or two-way ANOVA (G) followed by Tukey’s test. *p<0.05, ** p<0.01, ***p<0.001; **** p<0.0001 (Related to Fig. 3).

**Supplementary Material 5: Figure 5.** ABCA1 and caveolin1 expression in different cell types isolated from adult mouse brains. (A) Mouse brain tissues were homogenized with TBS and TBS containing 1% Triton X-100 (TBSX) and then dissolved with guanidinium chloride (GnHCl). Validation of ABCA1 and caveolin-1 expression in the different fractions. (B) Diagram of the isolation of different cell types from mouse brains. (C) Brains of APOE3 or APOE4-TR mice (male, 9-10-month-old) were collected and dissociated to form single-cell suspensions to isolate different cell types. Cell type-specific markers were used to determine the purity of the cell population using western blotting. ABCA1 and caveolin-1 protein levels were measured by western blotting. (D-E) Quantification of ABCA1 (D) and caveolin-1 expression (E) in different cell types (4 mice total from two independent experiments). Data are presented as mean± SD and were analyzed using one-way ANOVA followed by Tukey’s test. *p<0.05, **p<0.01, ****p<0.0001 (Related to Fig. 3).

**Supplementary Material 6: Figure 6.** Secondary only controls for p21 (A) and ABCA1-LAMP1 (B) immunostainings in mouse brain slides (Related to Fig. 4).

**Supplementary Material 7: Figure 7.** Effects of cyclodextrin treatment on body weight, cholesterol levels in APOE4-TR mice. (A) Body weight of mice in the experimental processing (PBS group, n=14, 5 females and 9 males; HPCD group n=15, 5 females and 10 males). (B) Total cholesterol level in plasma was measured by cholesterol assay kit. n=10 mice, 5 females and 5 males in each group. (C) Validation of lysosome isolation from mouse brain. Ponceau S staining (left panel) and immuno-blotting (right panel) were shown. (D-F) HDL particles in mouse CSF and plasma were analyzed by ion mobility. Concentration of HDL of different sizes in CSF (E) and plasma (F). (n=4-7 in each group). Data are represented as mean± SD (Related to Fig. 5).

**Supplementary Material 8: Figure 8.** Cyclodextrin increases neuronal functional markers and decreases cytokine levels in the brain of APOE4-TR mice. (A) Cytokines in the mouse cortex homogenate with TBSX buffer were measured using proteome profiler cytokine array kit, panel A. (n=2 samples each group, pooled 5 mice in one sample). (B) Synaptophysin levels in the hippocampus were determined using immunofluorescence staining (n=10). (C) PSD95, synaptophysin, and VGluT1 protein levels in the cortex were determined by western blotting (n=10). Data are presented as mean± SD and were analyzed using unpaired t-test. *p<0.05, **p<0.01, ****p<0.0001 (Related to Fig. 6).

**Supplementary Material 9: Figure 9.** Reduction of cholesterol by cyclodextrin reduces neuroinflammation in human iPSC-astrocytes model. (A)Validation of APOE4 human iPSC-astrocytes differentiation. (B) APOE4 human iPSC-astrocytes were cultured and loaded with LDL (10μg/mL) for 24 hours, followed by treatment with MβCD (1mM) for 2 hours. Cholesterol levels were measured using filipin staining. (C) After loading with LDL and treatment with MβCD, the cells were stimulated with TNFα+IFNγ for 18 hours. The mRNA levels of cytokines and chemokines were detected using qPCR (n=3 biological replicates). Data are presented as mean± SD and were analyzed using unpaired t-test. *p<0.05.

Supplementary Material 10

## Data Availability

Data are available from the corresponding author upon request. Analytical codes can be accessed by emailing the corresponding author. Sharing data from ROSMAP will follow ROSMAP data-sharing policies.

## Abbreviations

AD: Alzheimer’s disease
APOE: Apolipoprotein E
ROSMAP: Religious order study/memory aging project
SASP: Senescence-associated secretory phenotype
ABCA1: ATP-binding cassette A1
sn-RNA seq: Single-nucleus RNA sequencing
iPSC: Induced pluripotent stem cells
APOE-TR: APOE-target replacement
DLPFC: Dorsolateral prefrontal cortex
NCI: No cognitive impairment
MCI: Mild cognitive impairment
ssGSEA: Single-sample Gene Set Enrichment Analysis
CERAD: Consortium to Establish a Registry for Alzheimer’s Disease
mTOR: Mammalian target of rapamycin
mTORC1: mTOR complex 1
NFT: Neurofibrillary tangles
STG: SenTraGor
GFAP: Glia fibrillar acid protein
Iba1: Ionized calcium-binding adapter molecule 1
rE3/rE4: Recombinant ApoE3/ApoE4
GFP: Green fluorescent protein
LC-MS/MS: Liquid chromatography–mass spectrometry
SDS-PAGE: Sodium dodecyl-sulfate polyacrylamide gel electrophoresis
KEGG: Kyoto Encyclopedia of Genes and Genomes
DAVID: Database for Annotation, Visualization, and Integrated Discovery
Cav-1: Caveolin-1
AP2B1: Adaptor-related protein complex 2 subunit beta 1
BHK: Baby hamster kidney
HPCD: 2-hydroxypropyl-β-cyclodextrin
HDL: High-density lipoproteins
EEA1: Early endosome antigen 1
LAMP1: Lysosomal-associated membrane protein 1
NF-kB: Nuclear factor kappa-light-chain-enhancer of activated B cells
MβCD: Methyl-β-cyclodextrin
LDL: Low-density lipoproteins
TNF-α: Tumor necrosis factor alpha
IFN-γ: Interferon gamma
vGlut1: Type 1 vesicle glutamate transporter
PSD95: Postsynaptic density protein 95
NOR: Novel object recognition
A.U.: Arbitrary unit
WB: Western blotting
ROI: Region of interest
