# Supplementary figures and images for: Cellular senescence induced by cholesterol accumulation is mediated by lysosomal ABCA1 in APOE4 and AD
Source: Mol Neurodegener. 2025 Feb 4;20:15. doi: 10.1186/s13024-025-00802-7 (PMC11792374; doi:10.1186/s13024-025-00802-7)

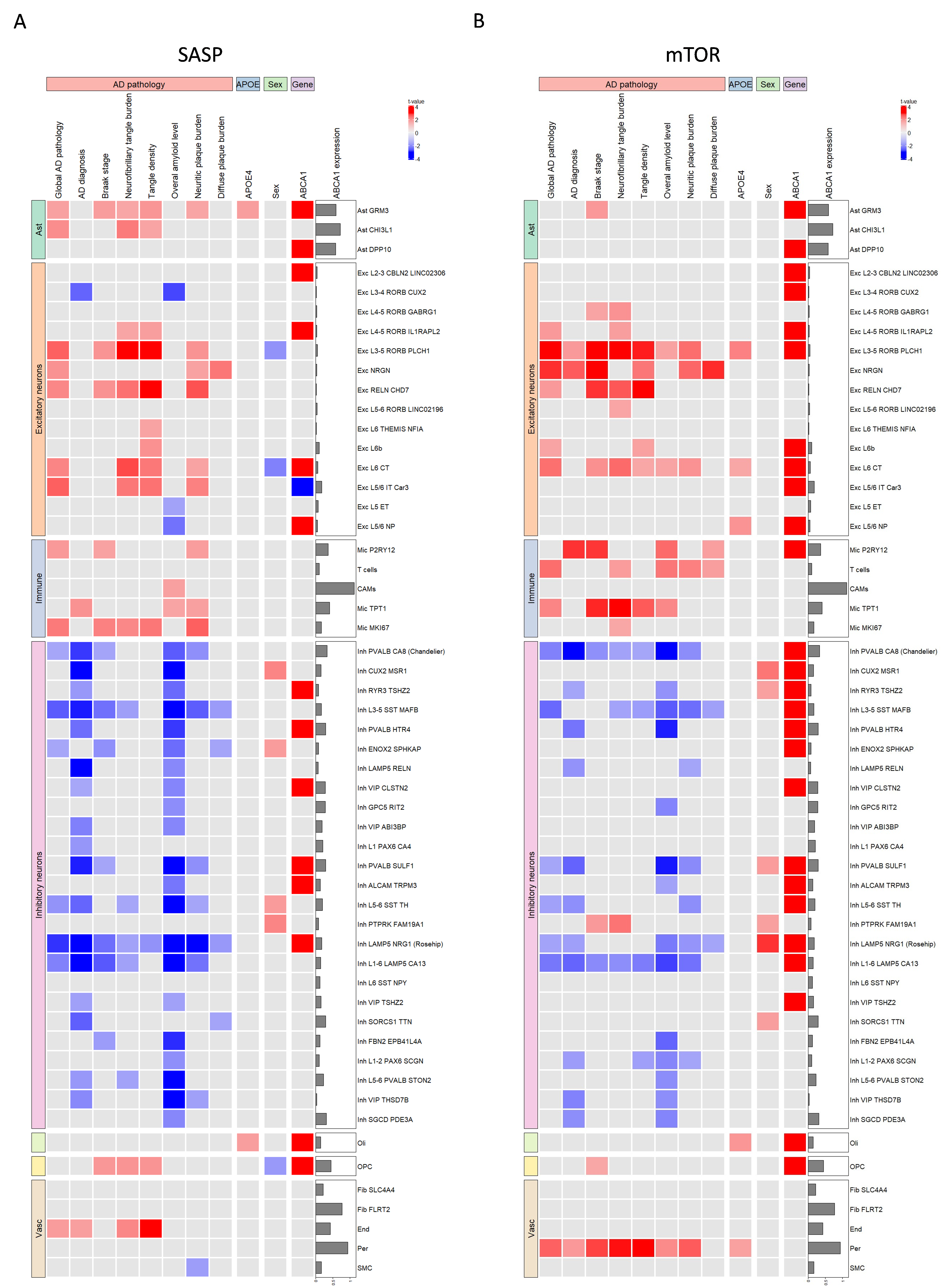

Supplement: Supplementary file 1 — Supplementary Material 1: Figure 1. Single-nucleus RNA (sn-RNA) sequencing analysis of the association between senescence-associated secretory phenotype (SASP) (A) and mTOR expression (B) in different brain cells with factors including AD pathology, sex, APOE4 genotype, and ABCA1 expression (Related to Fig. 1). [file 13024_2025_802_MOESM1_ESM.tif]

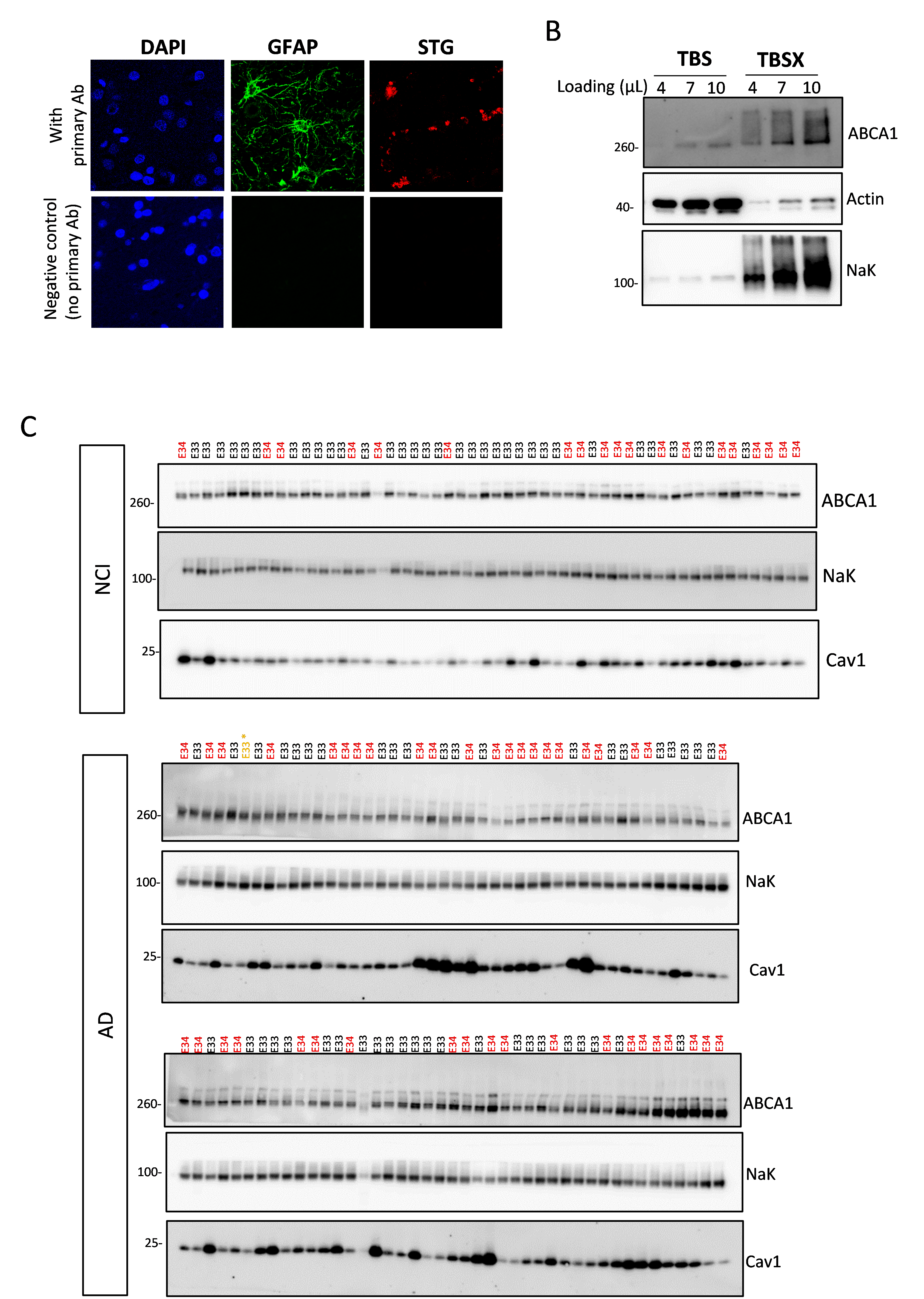

Supplement: Supplementary file 2 — Supplementary Material 2: Figure 2. Validation of immunofluorescent staining and whole blotting of western blots. (A) Validation of STG and GFAP staining. The negative control refers to omitting the primary antibody. (B) Frozen human postmortem mid-frontal lobe tissues were homogenized with different buffers and ABCA1 expression was detected by WB. Beta-actin and Na +/K + ATPase were used as markers for different fractions. (C) Total membrane ABCA1 and caveolin-1 protein levels in human postmortem mid-frontal lobe tissues were detected by WB. (NCI E3/3, n=33; NCI E3/4, n=19; AD E3/3, n=44; AD E3/4, n=42. One sample labelled with a star was excluded as it had MCI) (Related to Fig. 2). [file 13024_2025_802_MOESM2_ESM.tif]

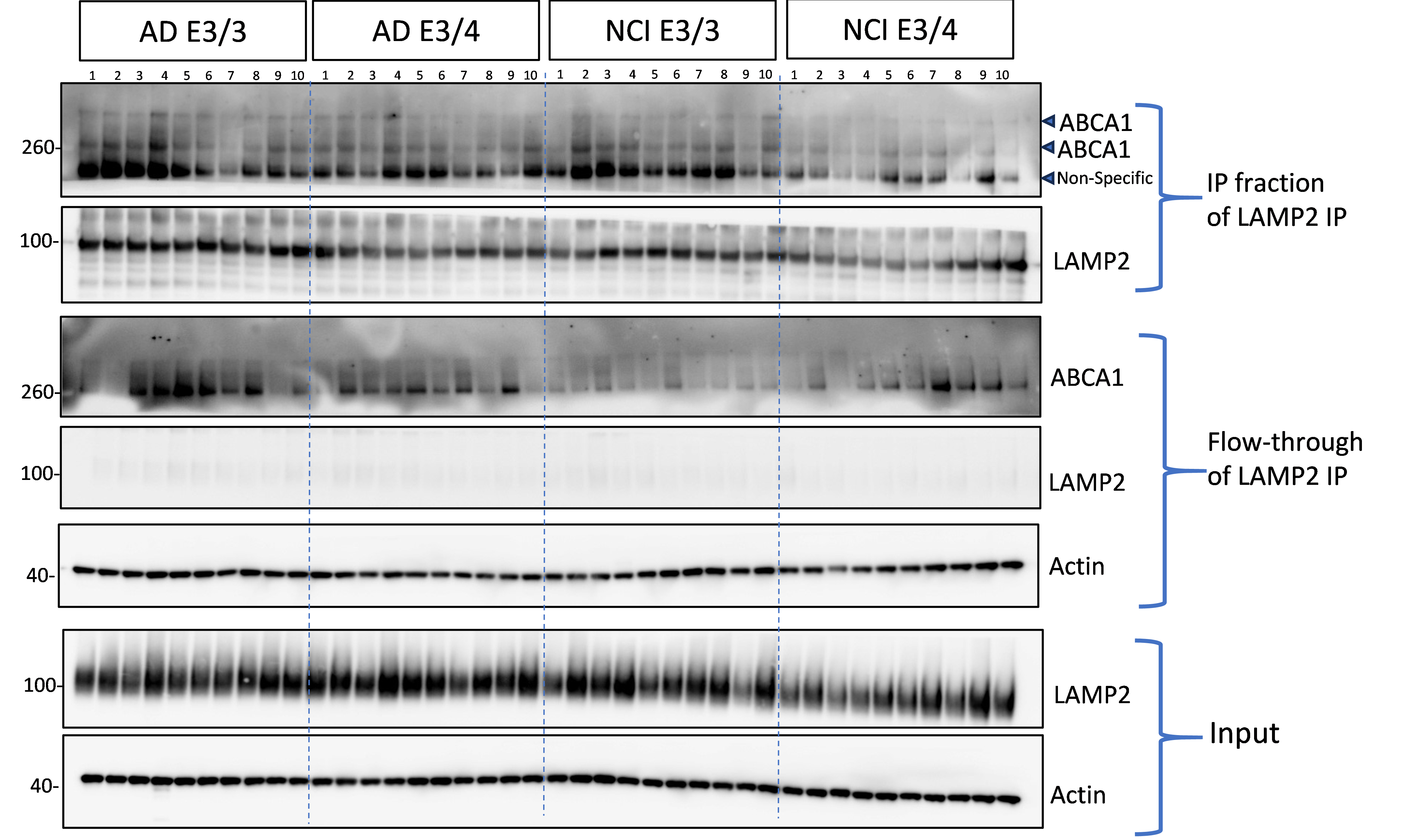

Supplement: Supplementary file 3 — Supplementary Material 3: Figure 3. The full blot of ABCA1 detection in lysosome membrane enriched fraction in human brain homogenate (immunoprecipitation with anti-LAMP2 antibody) (Related to Fig. 2C). [file 13024_2025_802_MOESM3_ESM.tif]

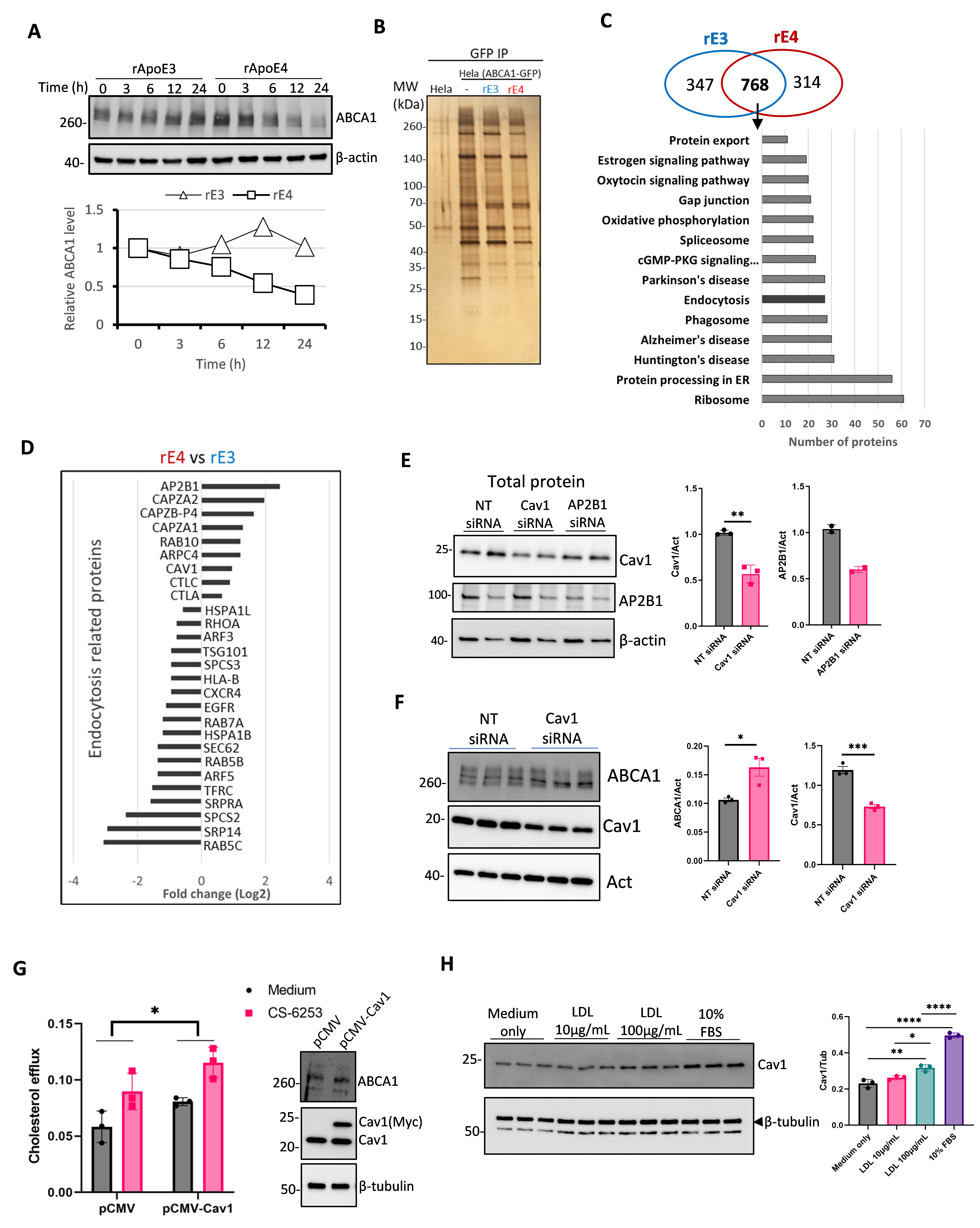

Supplement: Supplementary file 4 — Supplementary Material 4: Figure 4. Caveolin-1 regulates ABCA1 degradation. (A) HeLa cells expressing ABCA1-GFP were treated with recombinant ApoE3 or ApoE4 (0.2μM) for different hours. Cells were lysed with RIPA buffer and total ABCA1 levels were detected by WB using an anti-GFP antibody. The lower panel shows the densitometric quantification of the blots shown in the upper panel. ABCA1 was normalized to β-actin and then normalized to rApoE3 at the 0 h point. (B) Immunoprecipitation was performed with GFP-trap agarose in the lysate of HeLa cells or HeLa (ABCA1-GFP) cells treated with or without recombinant APOE3 (rE3) or APOE4 (rE4) for 4 h. Elution was detected using sodium dodecyl sulfate-polyacrylamide gel electrophoresis with silver staining. (C) 1115 and 1082 ABCA1 binding targets were detected in the recombinant APOE3 (rE3) and recombinant APOE4 (rE4) treatment groups, respectively, whereas 768 targets were found in both rE3 and rE4 groups. The top 14 candidates of the enriched KEGG pathways were identified from these 768 common targets (FDR < 0.05). (D) Enrichment levels (rE4 vs. rE3) of targets in the endocytic pathway. NSAF was used to calculate the fold change of each target. See the methods for calculating the NSFA. (E) Primary astrocytes were transfected with 15nM of non-target (NT), Caveolin-1 and AP2B1 siRNA for 48 h. The total protein levels of caveolin-1 and AP2B1 were detected by WB after siRNA transfection. (F) Immortalized astrocytes were transfected with 20nM of non-target (NT) or caveolin-1 siRNA for 48 h. The total protein levels of ABCA1 and caveolin-1 were detected by WB after siRNA transfection (n=3 biological replicates). (G) Caveolin-1 is overexpressed in immortalized astrocytes for 24 h, followed by labeling with 3H-cholesterol for 18 h. Cholesterol efflux was measured after treatment with the CS6253 peptides for 4 h (n=3 biological replicates). The right panel shows ABCA1 and caveolin-1 protein levels after plasmid transfection. (H) BHK [file 13024_2025_802_MOESM4_ESM.tif]

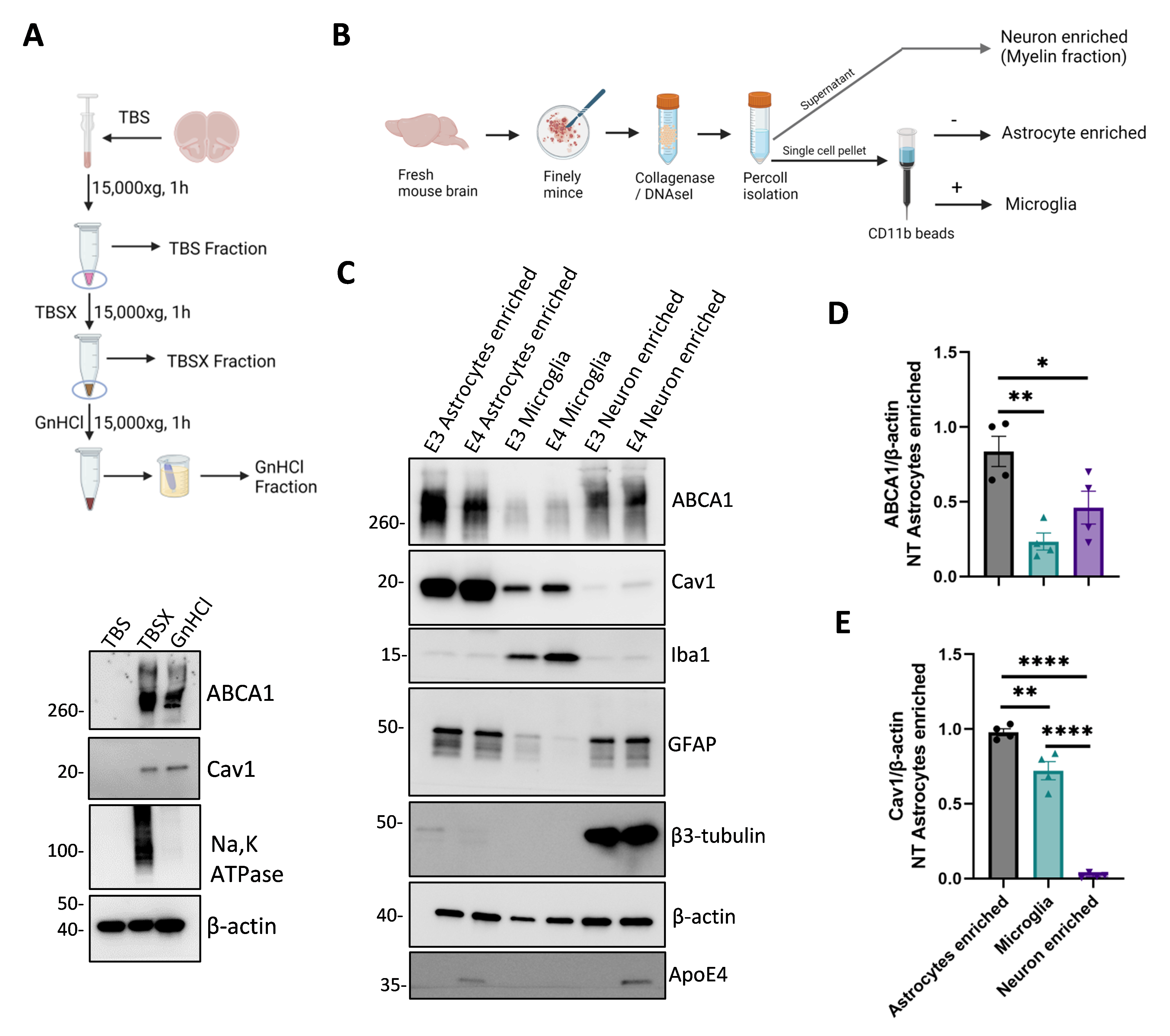

Supplement: Supplementary file 5 — Supplementary Material 5: Figure 5. ABCA1 and caveolin1 expression in different cell types isolated from adult mouse brains. (A) Mouse brain tissues were homogenized with TBS and TBS containing 1% Triton X-100 (TBSX) and then dissolved with guanidinium chloride (GnHCl). Validation of ABCA1 and caveolin-1 expression in the different fractions. (B) Diagram of the isolation of different cell types from mouse brains. (C) Brains of APOE3 or APOE4-TR mice (male, 9-10-month-old) were collected and dissociated to form single-cell suspensions to isolate different cell types. Cell type-specific markers were used to determine the purity of the cell population using western blotting. ABCA1 and caveolin-1 protein levels were measured by western blotting. (D-E) Quantification of ABCA1 (D) and caveolin-1 expression (E) in different cell types (4 mice total from two independent experiments). Data are presented as mean± SD and were analyzed using one-way ANOVA followed by Tukey’s test. *p<0.05, **p<0.01, ****p<0.0001 (Related to Fig. 3). [file 13024_2025_802_MOESM5_ESM.tif]

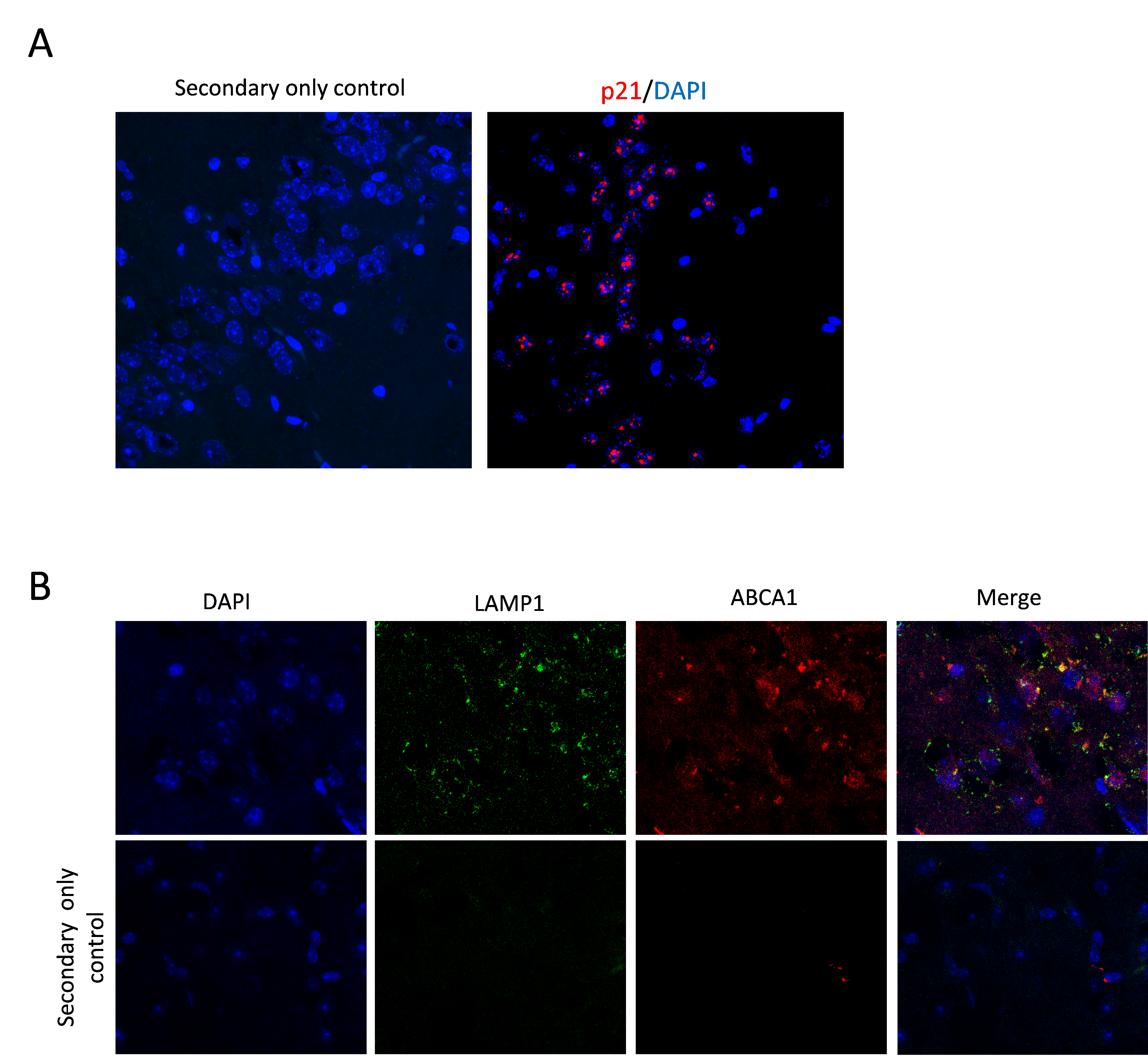

Supplement: Supplementary file 6 — Supplementary Material 6: Figure 6. Secondary only controls for p21 (A) and ABCA1-LAMP1 (B) immunostainings in mouse brain slides (Related to Fig. 4). [file 13024_2025_802_MOESM6_ESM.tif]

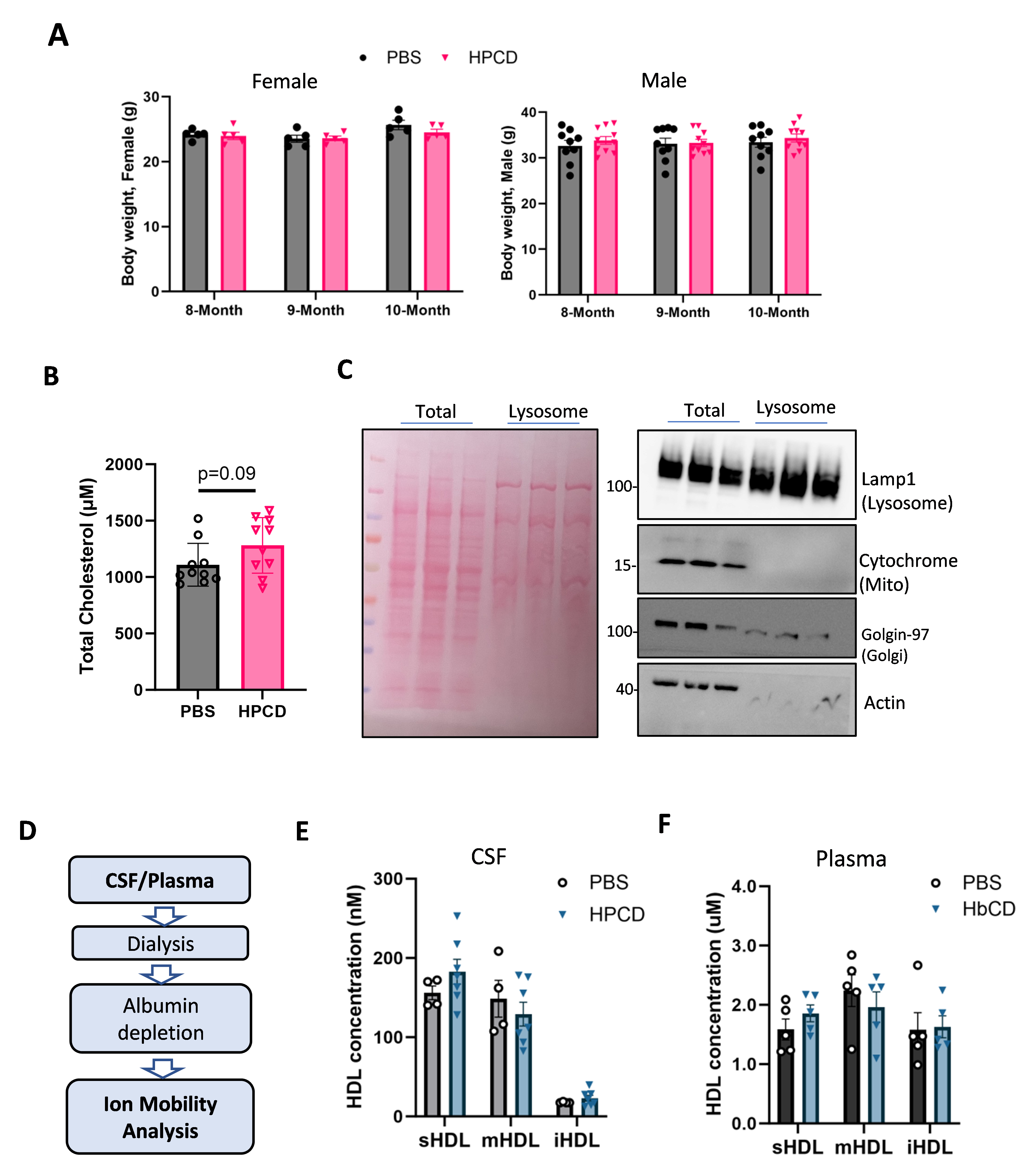

Supplement: Supplementary file 7 — Supplementary Material 7: Figure 7. Effects of cyclodextrin treatment on body weight, cholesterol levels in APOE4-TR mice. (A) Body weight of mice in the experimental processing (PBS group, n=14, 5 females and 9 males; HPCD group n=15, 5 females and 10 males). (B) Total cholesterol level in plasma was measured by cholesterol assay kit. n=10 mice, 5 females and 5 males in each group. (C) Validation of lysosome isolation from mouse brain. Ponceau S staining (left panel) and immuno-blotting (right panel) were shown. (D-F) HDL particles in mouse CSF and plasma were analyzed by ion mobility. Concentration of HDL of different sizes in CSF (E) and plasma (F). (n=4-7 in each group). Data are represented as mean± SD (Related to Fig. 5). [file 13024_2025_802_MOESM7_ESM.tif]

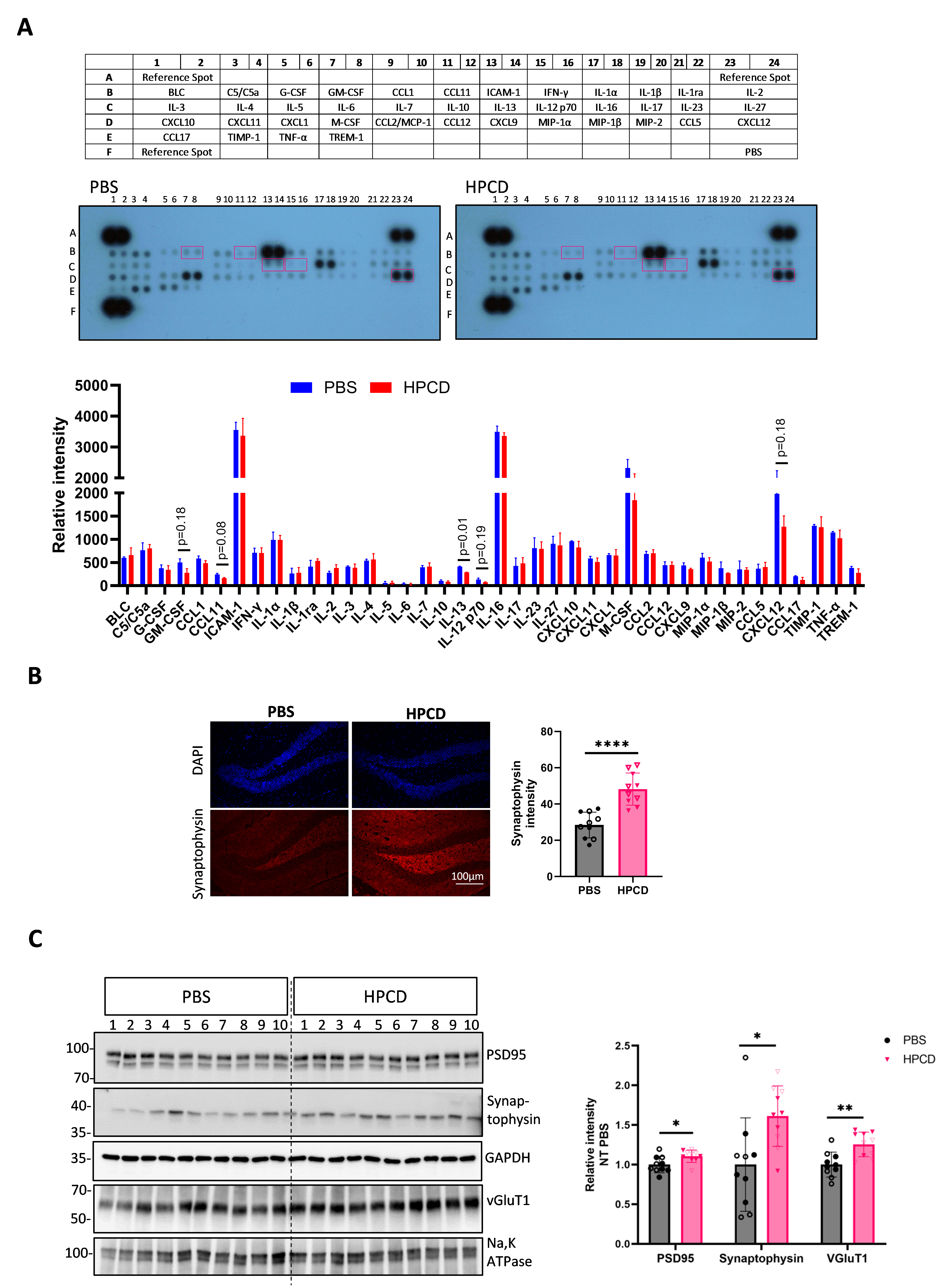

Supplement: Supplementary file 8 — Supplementary Material 8: Figure 8. Cyclodextrin increases neuronal functional markers and decreases cytokine levels in the brain of APOE4-TR mice. (A) Cytokines in the mouse cortex homogenate with TBSX buffer were measured using proteome profiler cytokine array kit, panel A. (n=2 samples each group, pooled 5 mice in one sample). (B) Synaptophysin levels in the hippocampus were determined using immunofluorescence staining (n=10). (C) PSD95, synaptophysin, and VGluT1 protein levels in the cortex were determined by western blotting (n=10). Data are presented as mean± SD and were analyzed using unpaired t-test. *p<0.05, **p<0.01, ****p<0.0001 (Related to Fig. 6). [file 13024_2025_802_MOESM8_ESM.tif]

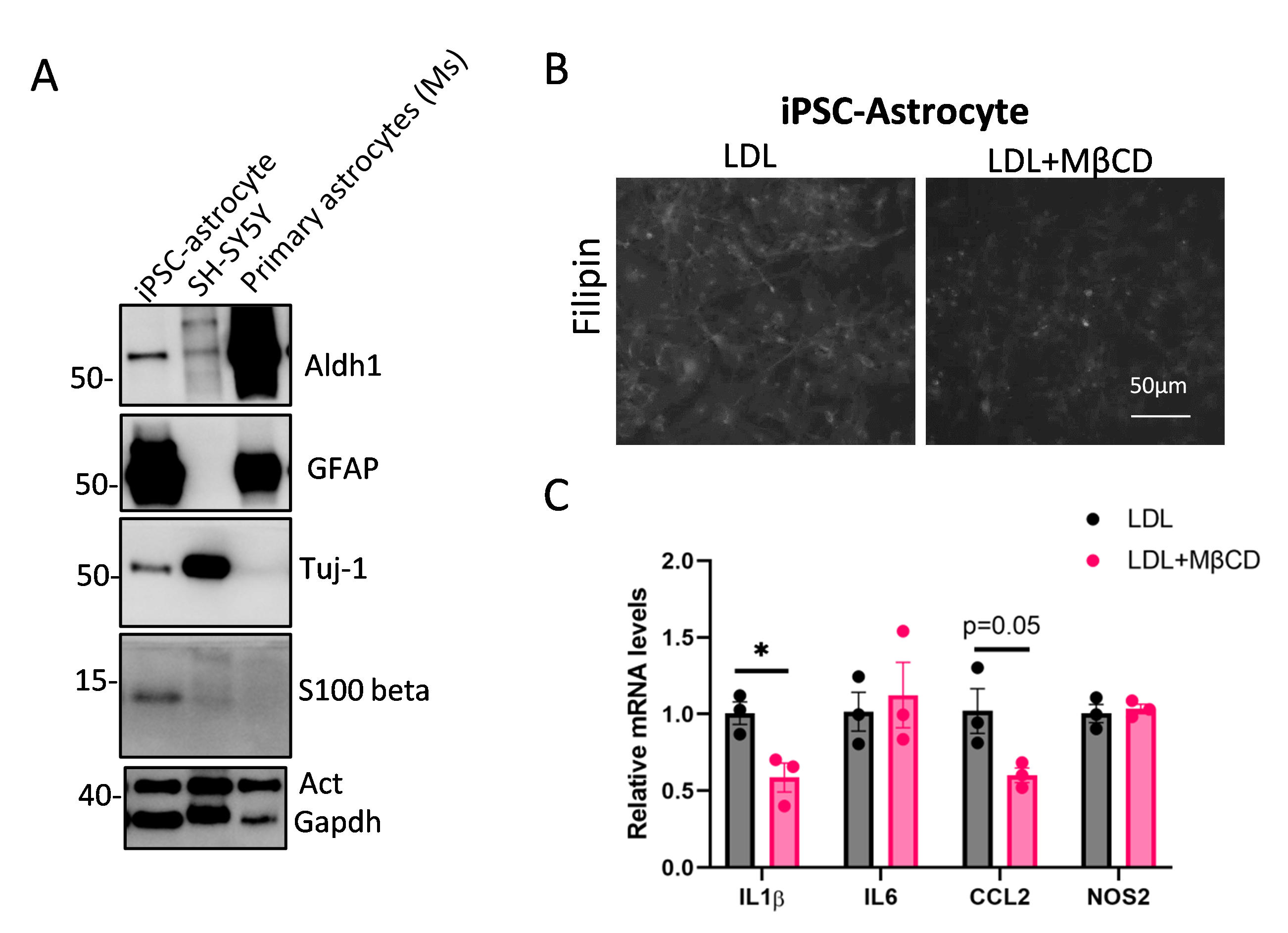

Supplement: Supplementary file 9 — Supplementary Material 9: Figure 9. Reduction of cholesterol by cyclodextrin reduces neuroinflammation in human iPSC-astrocytes model. (A)Validation of APOE4 human iPSC-astrocytes differentiation. (B) APOE4 human iPSC-astrocytes were cultured and loaded with LDL (10μg/mL) for 24 hours, followed by treatment with MβCD (1mM) for 2 hours. Cholesterol levels were measured using filipin staining. (C) After loading with LDL and treatment with MβCD, the cells were stimulated with TNFα+IFNγ for 18 hours. The mRNA levels of cytokines and chemokines were detected using qPCR (n=3 biological replicates). Data are presented as mean± SD and were analyzed using unpaired t-test. *p<0.05. [file 13024_2025_802_MOESM9_ESM.tif]
